# MicroRNA-375-3p Suppresses Upper Tract Urothelial Carcinoma Cell Migration and Invasion via Targeting Derlin-1

**DOI:** 10.3390/cancers14040880

**Published:** 2022-02-10

**Authors:** Jhen-Hao Jhan, Wei-Chi Hsu, Yi-Chen Lee, Wei-Ming Li, A-Mei Huang, Hui-Hui Lin, Chien-Sheng Wang, Yi-Ru Wu, Ching-Chia Li, Wen-Jeng Wu, Hung-Lung Ke

**Affiliations:** 1 Department of Urology, School of Medicine, College of Medicine, Kaohsiung Medical University, Kaohsiung 80708, Taiwan; ghostdeityj@gmail.com (J.-H.J.); westlife740205@gmail.com (W.-C.H.); u8401067@yahoo.com.tw (W.-M.L.); huihuilinkmu@yahoo.com.tw (H.-H.L.); ccli1010@hotmail.com (C.-C.L.); wejewu@kmu.edu.tw (W.-J.W.); 2 Department of Urology, Kaohsiung Medical University Hospital, Kaohsiung Medical University, Kaohsiung 80756, Taiwan; kcwang921@gmail.com; 3 Department of Urology, Kaohsiung Municipal Siaogang Hospital, Kaohsiung 81267, Taiwan; 4 Graduate Institute of Clinical Medicine, College of Medicine, Kaohsiung Medical University, Kaohsiung 80708, Taiwan; amhuang@kmu.edu.tw; 5 Graduate Institute of Medicine, College of Medicine, Kaohsiung Medical University, Kaohsiung 80708, Taiwan; 6 Department of Anatomy, School of Medicine, College of Medicine, Kaohsiung Medical University, Kaohsiung 80708, Taiwan; yichen83@kmu.edu.tw; 7 Department of Urology, Ministry of Health and Welfare, Pingtung Hospital, Pingtung 90054, Taiwan; 8 Department of Biochemistry, School of Medicine, College of Medicine, Kaohsiung Medical University, Kaohsiung 80708, Taiwan; 9 General Division, Kaohsiung Medical University Hospital, Kaohsiung Medical University, Kaohsiung 80708, Taiwan; k3329985@gmail.com; 10 Department of Urology, Kaohsiung Municipal Ta-Tung Hospital, Kaohsiung Medical University Hospital, Kaohsiung Medical University, Kaohsiung 80145, Taiwan

**Keywords:** upper tract urothelial carcinoma (UTUC), Derlin-1, miR-375-3p, epithelial–mesenchymal transition (EMT), biomarkers, prognostic factor, precision oncology

## Abstract

**Simple Summary:**

The Derlin-1 protein, encoded by *DERL1*, is located in the endoplasmic reticulum membrane and is responsible for the unfolded protein response. Derlin-1 has recently drawn lots of attention with regard to cancer pathogenesis. The purpose of this study is to examine its role and molecular mechanism in upper tract urothelial carcinoma (UTUC). The protein levels of Derlin-1 in human UTUC specimens are observed by immunohistochemistry. Derlin-1 overexpression is evidently associated with cancer stage, distant metastasis, recurrence, and poor prognosis in patients with UTUC. In an in vitro study, the knockdown of Derlin-1 represses, while over-expression of Derlin-1 increases migration and invasion of UTUC cells significantly. We identify the microRNA-375-3p (miR-375-3p) as one of the potential candidate microRNAs and then examine the relationship between Derlin-1 and miR-375-3p. The dual-luciferase reporter assay confirms that miR-375-3p directly targets Derlin-1. Furthermore, we reveal that miR-375-3p modulate Derlin-1 and epithelial–mesenchymal transition (EMT) marker protein levels. However, the above effects are reversed by the restoration of Derlin-1. Our study clarifies that miR-375-3p represses invasion and migration by directly targeting Derlin-1 and regulating the expression of EMT-associated proteins in UTUC cells, suggesting Derlin-1 may act as a useful predictor of prognosis and that both Derlin-1 and miR-375-3p could be potential therapeutic targets in UTUC.

**Abstract:**

Little is known regarding the molecular characterization of upper tract urothelial carcinoma (UTUC). Novel therapeutic targets and prognostic predictors are imminent. In the present study, we aim to examine the oncogenic function and molecular mechanism of Derlin-1 in UTUC. Derlin-1 overexpression is significantly associated with poor prognosis in patients with UTUC. In vitro, knockdown or over-expression of Derlin-1 markedly regulated UTUC cell invasion and migration. We further discovered miR-375-3p suppresses cell invasion and migration by inversely regulating Derlin-1 and blocking EMT in UTUC cells. Taking this together, miR-375-3p functions as a tumor suppressive microRNA by directly targeting Derlin-1 and blocking epithelial–mesenchymal transition (EMT) in UTUC.

## 1. Introduction

Upper tract urothelial carcinoma (UTUC) accounts for only 10% of all urothelial cancers worldwide, with a lack of available evidence on specific topics due to its rarity [1]. Surprisingly, the incidence of UTUC is exceptionally high in Taiwan, accounting for more than 30% of all urothelial cancers [2]. In Taiwan, UTUC is less common in men than in women, contrary to the male predominance in western countries [3]. In addition, higher grades and more advanced diseases were found in patients of Asian ethnicity compared to other ethnicities [4]. Over the past few decades, radical nephroureterectomy (RNU) has been considered the optimal treatment of choice for most patients with localized UTUC owing to the high proportion of high-grade tumors and advanced stages previously reported in large databases [5]. Even though considerable progress has been made in radiotherapy, chemotherapy, and novel immuno-oncology approaches to improve clinical outcomes, the overall survival of patients with UTUC remains unsatisfactory, which may result from some remarkable characteristics of urothelial carcinomas such as increased invasion and migration [6]. Because the prognosis of urothelial carcinoma depends on invasion depth and metastasis development, the exploration of additional novel molecules to inhibit the migration and invasion of urothelial carcinoma cells is deemed necessary [7].

MicroRNAs (miRNAs) are single-stranded noncoding RNAs that typically bind to the 3′-untranslated regions (3′-UTR) of the target messenger RNA (mRNA), regulating target gene expression either through translational suppression or mRNA degradation at the post-transcriptional level [8]. The dysregulation of miRNAs can result in various diseases, including malignancy [9]. During tumorigenesis, miRNAs can be regarded as tumor suppressor genes or oncogenes based on their multiple target mRNAs and varying downstream expression levels [10]. A complex network involving multiple miRNAs and their downstream effectors could mediate the malignant progression of different tumors, including non-small cell lung cancer [11], breast cancer [12], and acute myeloid leukemia [13]. The diagnostic and prognostic significance of miRNAs has been validated in head and neck cancer [14], colorectal cancer [15], and neuroendocrine tumors [16]. MiR-375-3p and miR-133a-3p are significantly down-regulated in patients with oral cancer and can be used as early diagnostic biomarkers [17]. In patients with UTUC, the prognostic value of miRNAs has also been validated [18], which may have promising implications for making clinical decisions [19].

Degradation in endoplasmic reticulum protein 1 (Derlin-1) was an endoplasmic reticulum (ER) membrane protein. Derlin-1 is translated from the human gene *DERL1* situated on chromosome 8q and is reportedly a multifunctional protein that comprises four transmembrane domains, with both a C-terminus and an N-terminus within the intracellular fluid [20]. Derlin-1 is mainly responsible for modulating the elimination of misfolded proteins from the ER and the retro-translocation of proteins into the cytoplasmic matrix [21]. Recently, emerging studies have implied the role of Derlin-1 in carcinogenesis and tumor progression. An elevated expression level of Derlin-1 may correlate with the malignant behavior of several cancer types, including liver cancer [22], breast cancer [23], lung cancer [24], head and neck cancer [25], and colon cancer [26]. Additionally, Derlin-1 is overexpressed and correlated with aggressive behavior in muscle-invasive bladder urothelium carcinoma [27]. The role of miRNA in the molecular pathway in non-small cell lung cancer has been investigated by negatively regulating Derlin-1 [28]. However, the correlation between Derlin-1 and miRNAs has not yet been elucidated in UTUC. In this study, we purposed to explore the oncogenic function and molecular mechanism of Derlin-1 and miRNAs in UTUC. We identified miR-375-3p as one of the potential candidate miRNAs that functions by directly binding to the 3′-UTR of Derlin-1, regulating epithelial–mesenchymal transition (EMT) and controlling cell invasion and migration in UTUC cells.

## 2. Materials and Methods

### 2.1. Clinical Tissues and Clinicopathological Data

The research was approved by the Institutional Review Board of the Kaohsiung Medical University (KMUHIRB-G(I)-20190037). The specific method is stated in our previous study [29].

All enrolled patients signed consents and received nephroureterectomy and bladder cuff removal. We retrospectively inspected all patients’ medical records and retrieved clinicopathological data. The median follow-up time was 40.4 months. Return visits were generally performed every three months until two years after surgery and at increasing intervals thereafter. Follow-up visits consisted of a routine investigation, physical examination, and serum chemistry profile analysis. Cystoscopy was performed every three months until two years after surgery and then at increasing intervals to check for bladder recurrence. The follow-up interval of the images, including chest radiography and echography or computed tomography of the abdomen and pelvis, is based on the National Comprehensive Cancer Network (NCCN) guidelines and clinician’s preference.

### 2.2. Immunohistochemical Analysis of Derlin-1

The slides were incubated with a 1:150 dilution of an anti-DERL1 polyclonal antibody (HPA016562, Atlas Antibodies, Sweden). Negative control means tissue slides incubated with antibody diluted solution without Derlin-1 antibody. Secondary antibodies were incubated using the Dako REAL EnVision Detection System (K5007, Dako, Carpinteria, CA, USA). Finally, the slides were counterstained with hematoxylin and observed by light microscopy. The specific method is stated in our previous study [29].

### 2.3. Estimation of Immunohistochemical Staining

Scoring for Derlin-1 staining was determined based on the percentage of positively stained cells in two quantitative categories. Any dissimilarities in scoring between the pathologists were jointly reviewed, and a concordance was reached. As a representation of indicative Derlin-1 expression, tumors with <50% positively stained cells were categorized as low levels, whereas tumors with >50% positively stained cells were ranked as high levels. The specific method is stated in our previous study [29].

### 2.4. Cell Culture

In this study, we used the following three cell lines: BFTC909, UM-UC-14, and SV-HUC-1 cells. The specific method is stated in our previous study [29].

### 2.5. RNA Isolation and Real-Time PCR Analyses

The following primers were used in the real-time PCR experiment: *DERL1* sense, 5′-CAATGGACTTGGGAGGAAGAAA-3′; *DERL1* antisense 5′-CTAGCAGGGGGCACACCA-3′; *GAPDH* sense, 5′-GCACCACCAACTGCTTAGCA-3′; and *GAPDH* antisense, 5′-TCTTCTGGGTGGCAGTGATG-3′. The relative levels of *DERL1* mRNA are expressed as the inverse log of the ΔΔCt value and normalized with the internal control gene *GAPDH*. The relative levels of miR-375-3p are expressed as the inverse log of the ΔΔCt levels and normalized with the internal control gene, U6 snRNA. The specific method is stated in our previous study [29].

### 2.6. Cell Transfection

UTUC cells were transfected with hsa-miR-375-3p mimics (50 nm), negative control (50 nm) of miRNA, CMV-DERL1 (6 ug) (Sino Biological, Wayne, PA, USA) or DERL1 Silencer Select siRNA (10 nm) (s35607, Thermo Fisher Scientific, MA, USA). According to the manufacturer’s protocol, cell transfections were performed using TurboFect (Thermo Scientific, MA, USA). The specific method is stated in our previous study [29].

### 2.7. Western Blot Analysis

The specific method is stated in our previous study [29]. Membranes were incubated overnight at 4 °C with anti-Derlin-1 (HPA016562, Atlas Antibodies), β-Actin (#3700, Cell Signaling Technology, MA, USA), α-tubulin (NB100-690, Novus, Littleton, CO, USA), E-cadherin (NBP2-19051, Novus, Littleton, CO, USA), N-cadherin (#13116, Cell Signaling Technology, MA, USA), vimentin (#3390, Cell Signaling Technology, MA, USA), Snail (NBP2-27184, Novus, Littleton, CO, USA), MMP2 (GTX30147, GeneTex, Alton Pkwy Irvine, CA, USA), occludin (CB1044, Sigma-Aldrich, Burlington, MA, USA), and ZEB1 (#3396, Cell Signaling Technology, MA, USA).

### 2.8. Cell Proliferation Assay

Cell proliferation was examined by WST-1 (Takara Bio, Mountain View, CA, USA). The specific method is stated in our previous study [29].

### 2.9. Wound Healing Assay

Wound healing assay was used by 2-well culture-inserts (ibidi, Gräfelfing, Germany). UTUC cells were counted and seeded in inserts after transfection. After 24 h of incubation, the insert was removed to create the gap. The gap of UTUC cells was photographed at different time points using a phase-contrast microscope. The specific method is stated in our previous study [29].

### 2.10. Cell Invasion Assays

Cell invasion was analyzed by ECMatrix cell invasion assay (Merck, Darmstadt, Germany). The specific method is stated in our previous study [29].

### 2.11. Dual-Luciferase Reporter Assay

For dual-luciferase reporter assay, BFTC909 cells were seeded in a 96-well plate and co-transfected with pmirGLO dual-luciferase miRNA target expression vector (catalog number: E1330, Promega, Madison, WI, USA) using TurboFect reagent (Thermo Scientific, MA, USA). The samples were wild-type DERL1, mutant DERL1, or scramble control and miR-375-3p mimics. Renilla and Firefly luciferase activities were measured 48 h post-transfection using the dual-luciferase reporter assay kit (Promega, Madison, WI, USA) and Microplate Luminometer (BioTek, Winooski, VT, USA). The specific method is stated in our previous study [29].

### 2.12. Statistical Analysis

Experiments were presented at least three times independently. The results are shown as mean ± SD. The specific method is stated in our previous study [29]. The two-tailed t-test verified the statistical significance of dissimilarity between groups. *p* values < 0.05 were consider that significant.

## 3. Results

### 3.1. Clinical Significance of Derlin-1 in Patients of UTUC

**Figure 1:**
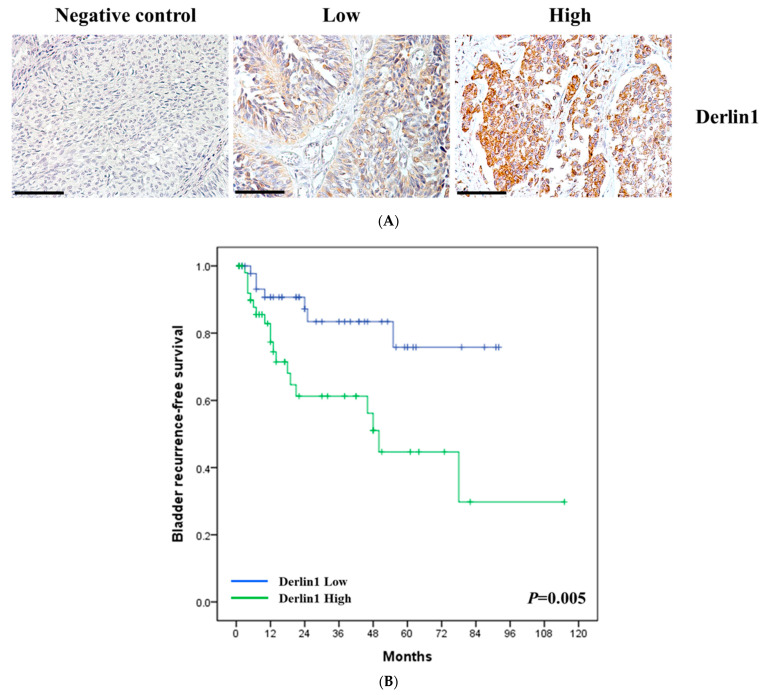

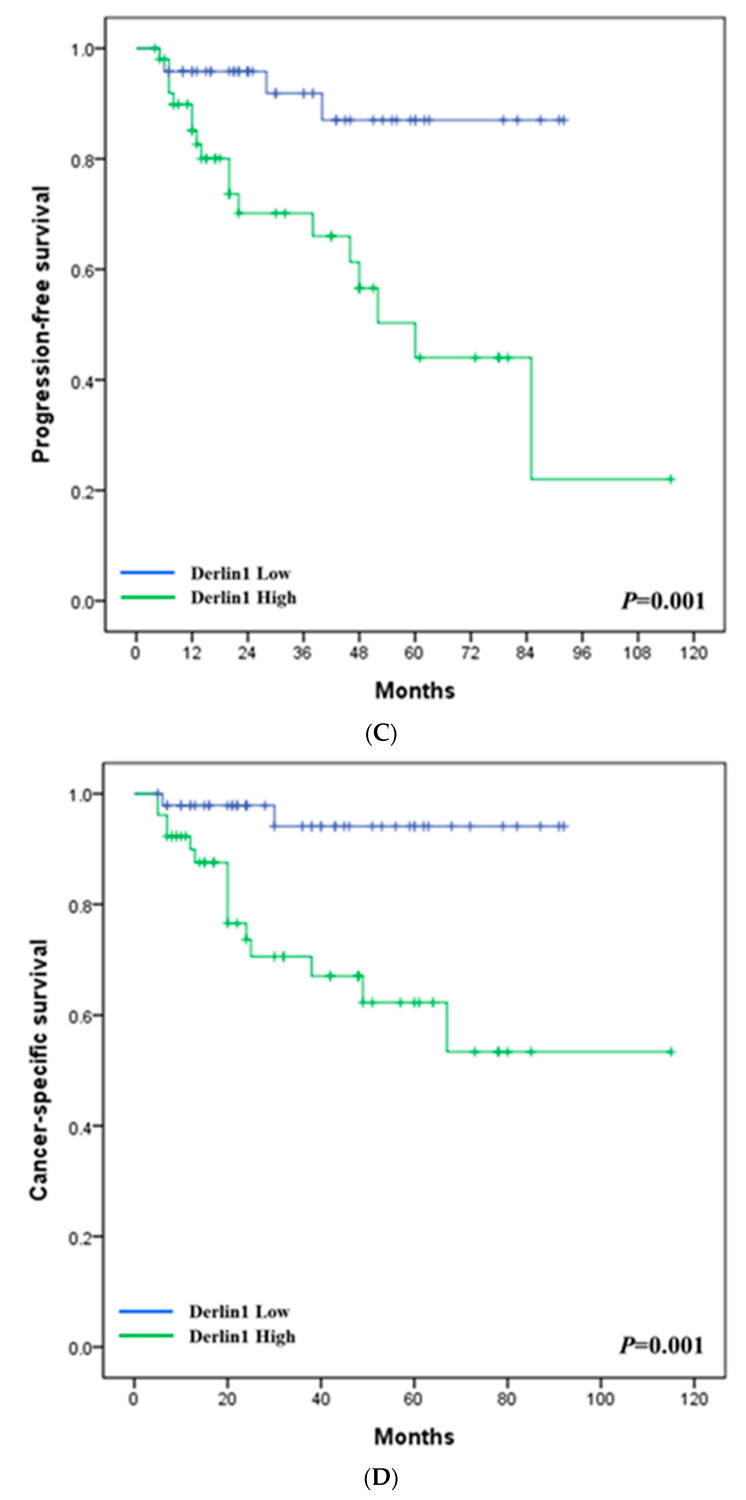
Derlin-1 is overexpressed in UTUC tissues and is associated with survival of patients. (**A**) The staining intensity of Derlin-1 of UTUC samples was examined by immunohistochemistry. The range of Derlin-1 expression partitioned into negative control, low and high. (**B**) Kaplan-Meier survival curves for bladder recurrence-free survival of patients of UTUC. (**C**) Kaplan-Meier survival curves for progression-free survival of patients of UTUC. (**D**) Kaplan-Meier survival curves for cancer-specific survival of patients of UTUC. Scale bar, 50 μm.

To investigate the clinical significance of Derlin-1 in UTUC, the protein expression levels of Derlin-1 in 100 UTUC tissue samples were identified as high and low levels on the basis of the intensity of cytoplasm staining of cancer cells using immunohistochemistry (Figure 1A). A negative control was also shown. Fifty-two (52%) tumor specimens had high levels of Derlin-1 staining, and forty-eight (48%) had low levels. We retrospectively collected important clinical and pathological characteristics, including gender and age of the patients; pathological stage, grade, size, number, and location of the tumors; the status of lymph node metastasis and distant metastasis in this cohort. A bladder recurrence, local recurrence, and cancer-specific death were also presented. A high Derlin-1 staining level was significantly correlated with the pathological stage (*p* = 0.019), the status of distant metastasis (*p* = 0.019), bladder recurrence (*p* = 0.012), tumor-related mortality (*p* = 0.001) and local recurrence (*p* = 0.002) (Table 1).

Next, the multivariate analyses revealed that high Derlin-1 staining level were significantly associated with bladder recurrence-free survival (HR = 3.62, *p* = 0.004; Table 2), progression-free survival (HR = 4.68, *p* = 0.007; Table 3), and cancer-specific survival (HR = 6.24, *p* = 0.017; Table 4). The pathological stage was also significantly correlated with progression-free survival (HR = 4.81, *p* = 0.004; Table 3) and cancer-specific survival (HR = 5.65, *p* = 0.004; Table 4). Furthermore, high-expression Derlin-1 had notably lower bladder recurrence-free survival (*p* = 0.005; Figure 1B), progression-free survival (*p* = 0.001; Figure 1C) and cancer-specific survival (*p* = 0.001; Figure 1D) than those with a low Derlin-1 expression level. These data suggest that Derlin-1 overexpression were able to predict poor prognosis in patients with UTUC.

### 3.2. Derlin-1 Is Overexpressed in UTUC Cell Lines and Enhances Migration and Invasion

**Figure 2:**
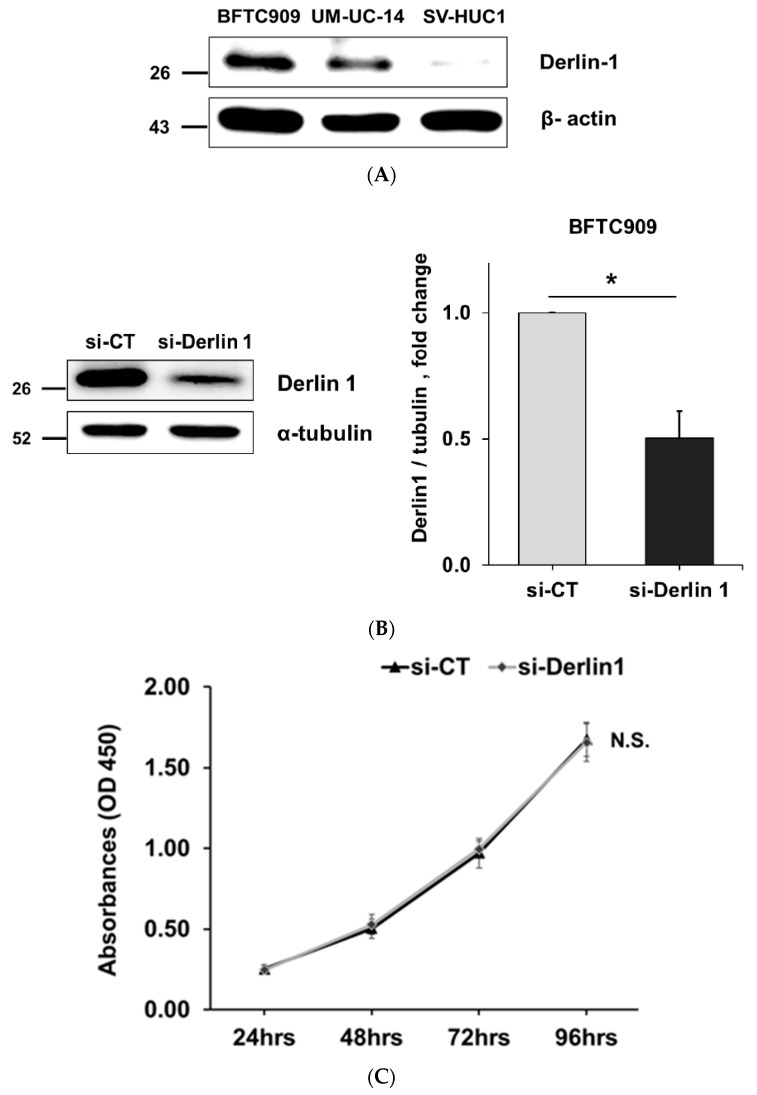

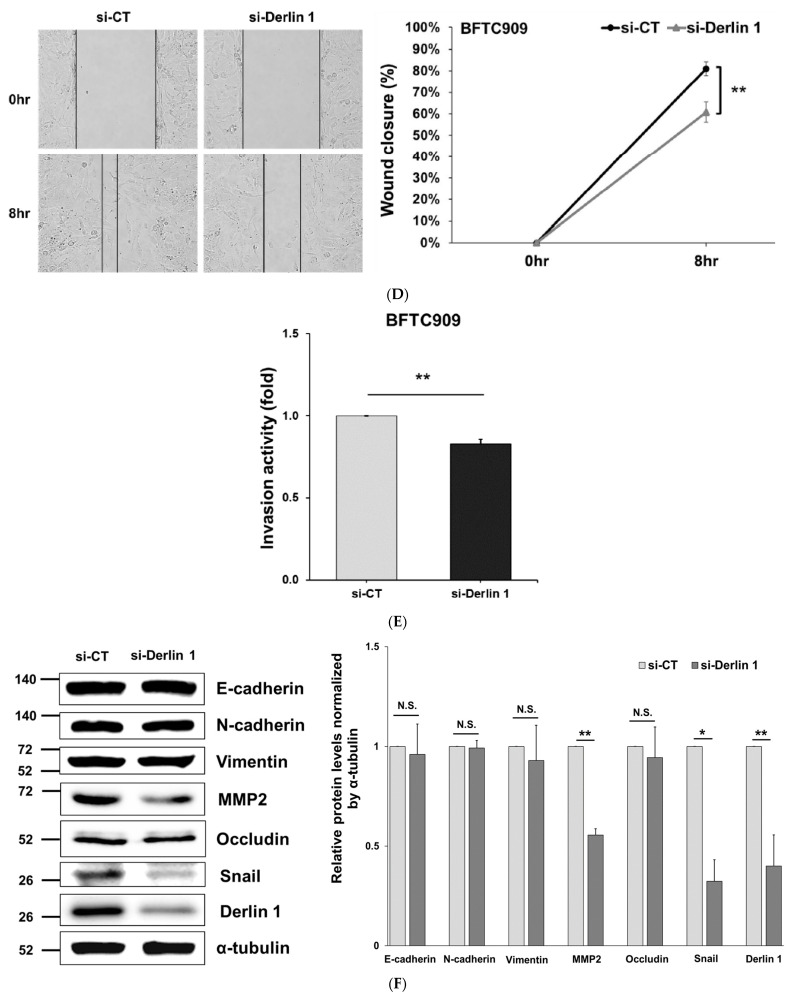

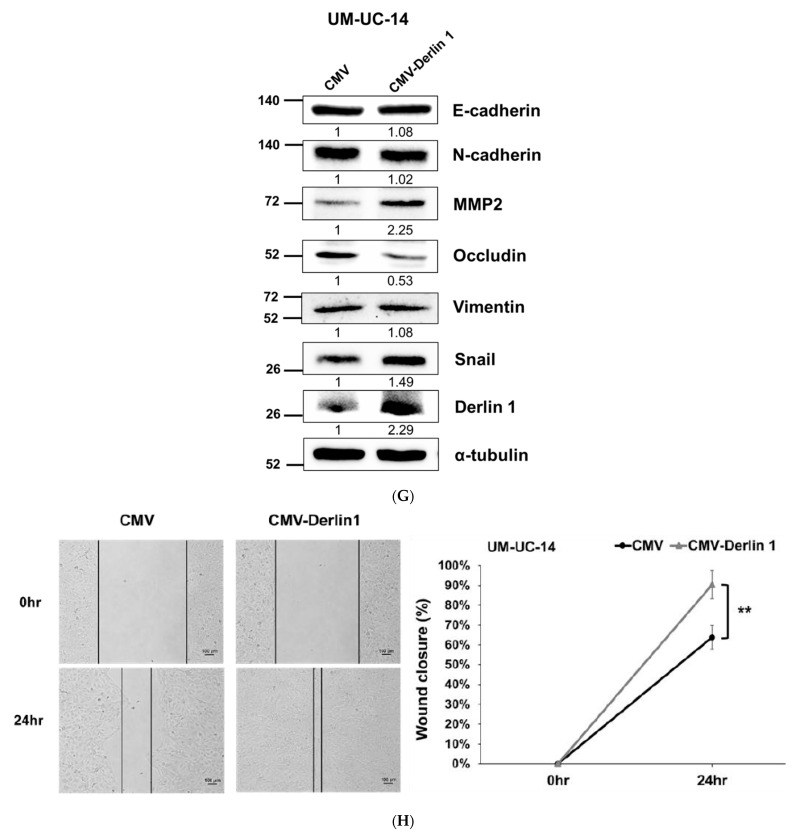

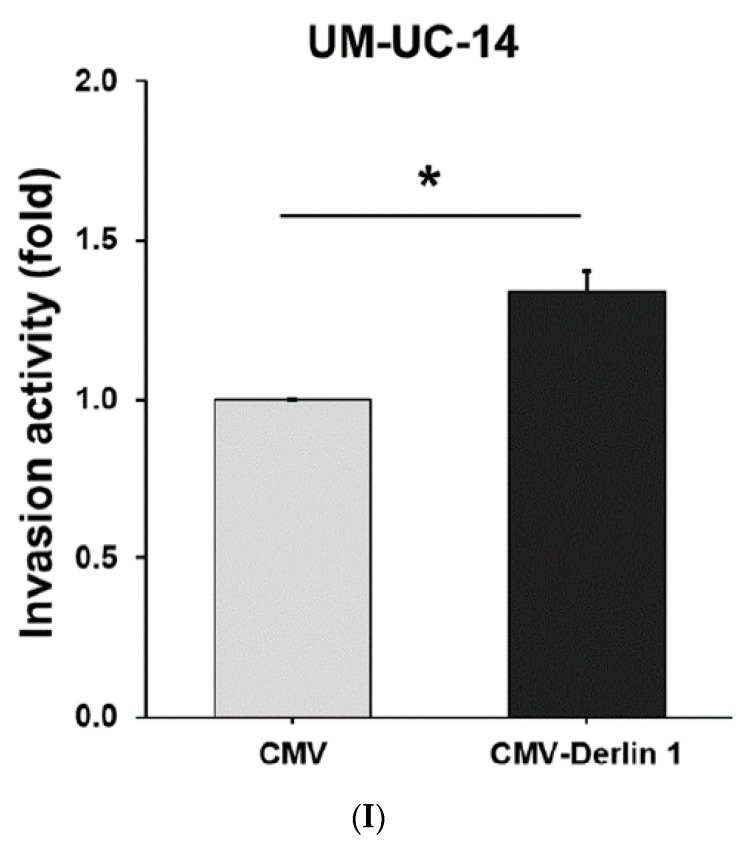
Derlin-1 is overexpressed in UTUC cell lines and enhances migration and invasion. (**A**) The Derlin-1 protein levels in cell by Western blot analysis with α-tubulin as a reference. Derlin-1 was highly expressed in BFTC909 and UM-UC-14 cells compared with SV-HUC-1 cells. (**B**) The Derlin-1 protein levels in BFTC909 cell lines by Western blot analysis after transfection with control siRNA (si-CT) or Derlin-1 siRNA (si-Derlin-1) as indicated (N = 3). (**C**) Knockdown Derlin-1 did not affect proliferation of BFTC909 cells (N = 3). (**D**) Knockdown Derlin-1 represses migration of BFTC909 cells (N = 3). (**E**) Knockdown Derlin-1 represses invasion of BFTC909 cells (N = 3). (**F**) The protein levels of well-known EMT markers were examined after knockdown of Derlin-1 in BFTC909 cells (N = 3). Knockdown Derlin-1 reduces the expression level of Snail and MMP2 in BFTC909 cells. The E-cadherin, N-cadherin, vimentin and occludin protein expression levels were not changed significantly. (**G**) Overexpression of Derlin-1 reduces the protein level of occludin and induces MMP2 and Snail in UM-UC-14 cells. The expression level of E-cadherin, N-cadherin was not changed significantly. (**H**) Overexpression of Derlin-1 enhances migration of UM-UC-14 cells. (**I**) Overexpression of Derlin-1 enhances invasion of UM-UC-14 cells. Data were represented as mean ± SD; * *p* < 0.05, ** *p* < 0.01. N.S: not significant. Full Western blot images can be found at Supplementary File S1.

BFTC909 and UM-UC-14 are UTUC cell lines. These cells had higher Derlin-1 protein levels compared with immortalized human normal urothelium cells (SV-HUC-1), especially in BFTC909 cells (Figure 2A). In addition, our previous study found that compared with UM-UC-14, BFTC909 had a more mesenchymal-like morphology [29]. Therefore, this study mainly focused on BFTC909 cells.

To examine the oncogenic roles of Derlin-1 in UTUC, we knocked down Derlin-1 via RNA interference in BFTC909 cells (Figure 2B). Cell proliferation was not affected by the silencing of Derlin-1 expression in BFTC909 cells (Figure 2C). However, the wound healing assay revealed a remarkable reduction in migration in Derlin-1-silencing cells (Figure 2D); Figure 2E also revealed the inhibitory effects on the invading ability of BFTC909 cells upon knocking down Derlin-1. Then, we examined the protein expression of some well-known EMT markers after the knockdown of Derlin-1 in BFTC909 cells. The expression level of MMP2 (matrix metallopeptidase-2) and Snail was significantly reduced (Figure 2F). Snail is a well-known activator or repressor of EMT [30,31]. In tumor cells, the protein level of Derlin-1 in UM-UC-14 cells was lower than in BFTC909 cells (Figure 2A), thus we overexpressed Derlin-1 in UM-UC-14 cells to observe whether it affected cell function. The data showed that Derlin-1 overexpression downregulated occludin and upregulated Snail and MMP2 protein expression in UM-UC-14 cells (Figure 2G). The overexpression of Derlin-1 resulted in the promotion of UM-UC-14 cell migration and invasion (Figure 2H,I).

These results suggest that Derlin-1 enhances cell invasion and migration of UTUC cells, which may be related to EMT.

### 3.3. MiR-375-3p Downregulates Derlin-1 Protein Expression via Directly Targeting Derlin-1 in BFTC909 Cells

**Figure 3:**
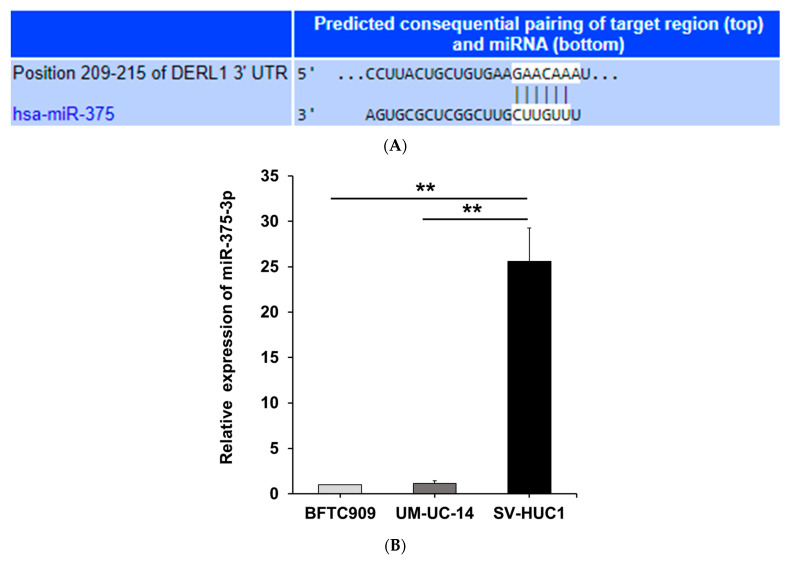

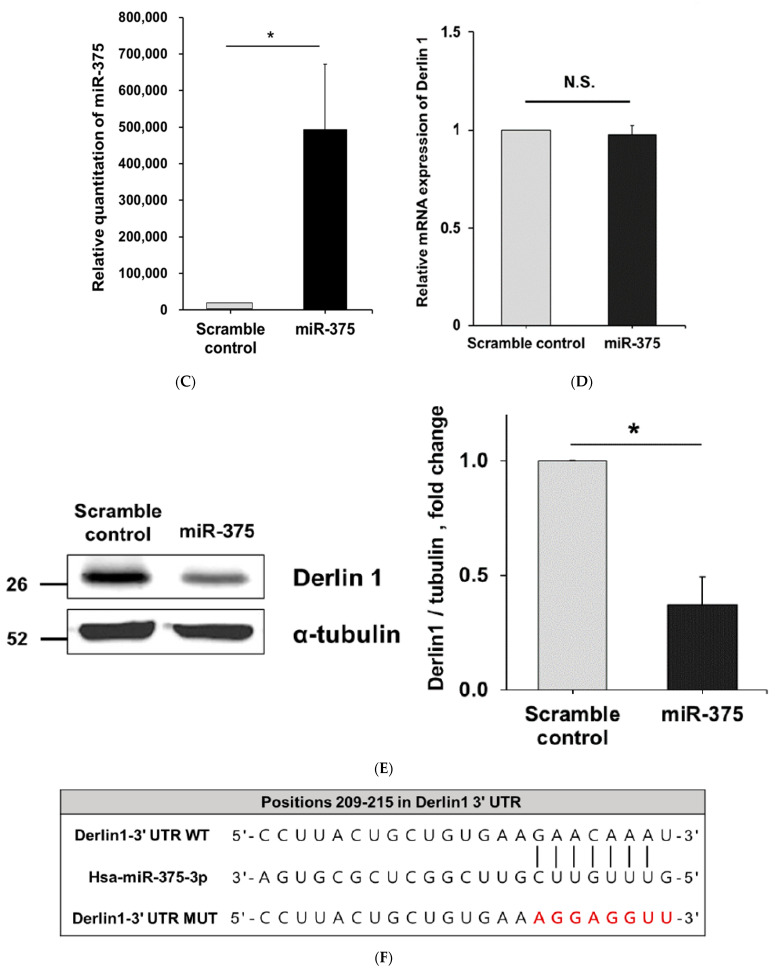

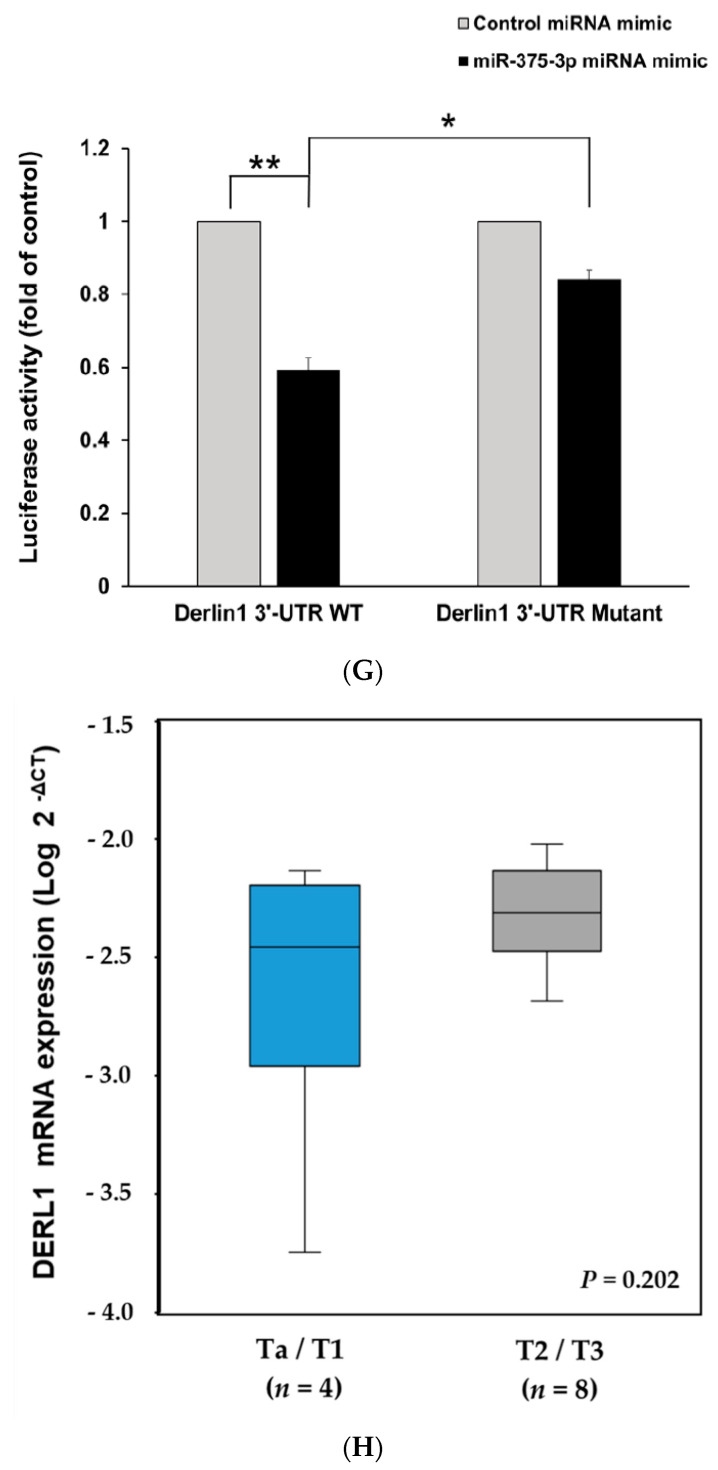

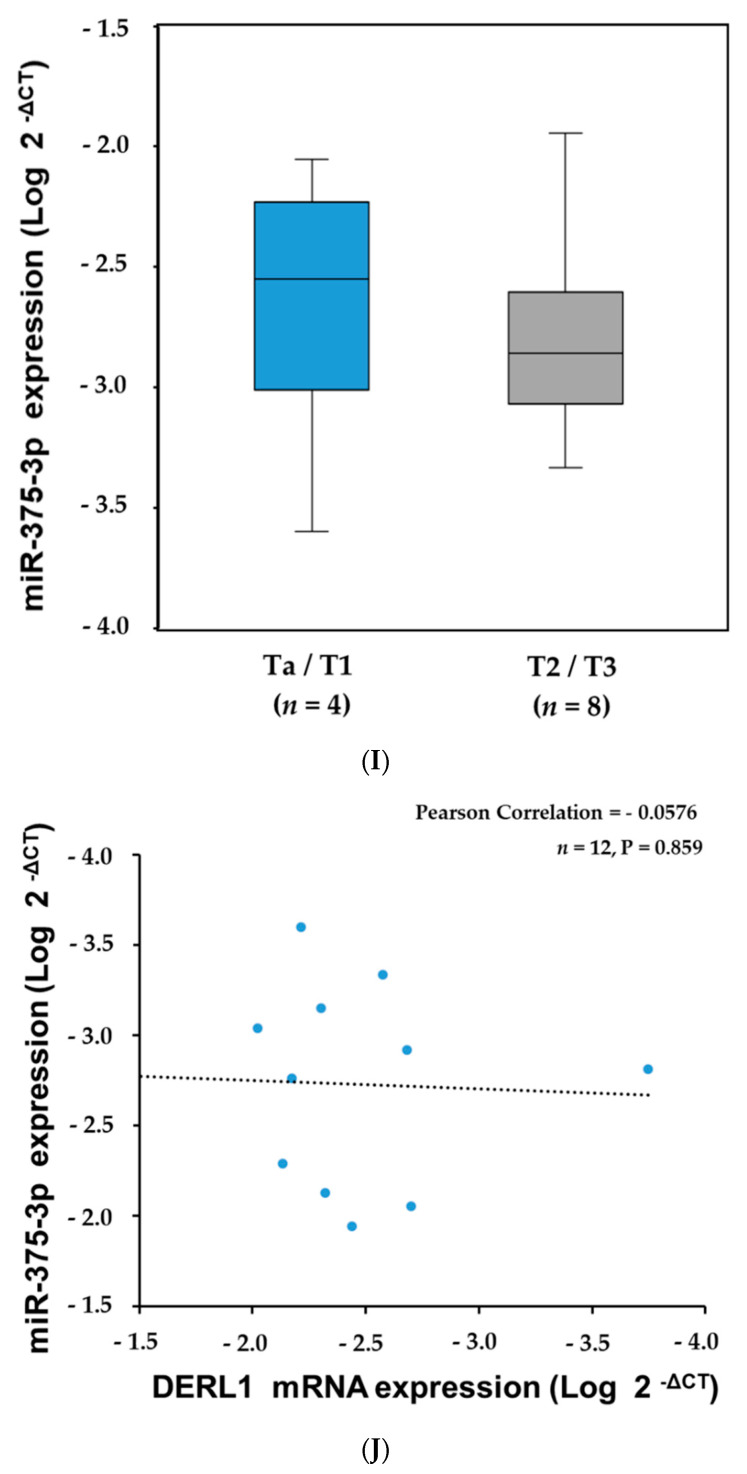
MiR-375-3p downregulates Derlin-1 protein expression via directly targeting Derlin-1 in UTUC cells. (**A**) miR-375-3p was identified as one of the potential candidate miRNAs from TargetScan 7.2 software. (**B**) BFTC909, UM-UC-14, and SV-HUC-1 cells were analyzed miR-375-3p expression by qRT-PCR (N = 3). (**C**) BFTC909 cells were analyzed miR-375-3p expression by qRT-PCR after transfected with scramble control or miR-375-3p mimics (N = 3). (**D**) Upregulation of miR-375-3p did not change the transcription of *DERL1* in BFTC909 cells (N = 3). (**E**) miR-375-3p overexpression reduced Derlin-1 protein levels in BFTC909 cells (N = 3). (**F**) The possible binding site of hsa-miR-375-3p and Derlin-1 predicted by TargetScan. (**G**) Dual-luciferase reporter assay demonstrated miR-375-3p directly binding to Derlin-1 in BFTC909 cells. (N = 3). (**H**–**J**) The expression of Derlin-1 mRNA and miR-375-3p in our tumor specimens were examined. Data were represented as mean ± SD; * *p* < 0.05, ** *p* < 0.01. N.S: not significant.

To elucidate the underlying mechanism of oncogenic Derlin-1 in promoting migration and invasion of UTUC, we used TargetScan 7.2 software (http://www.targetscan.org/vert_72/, accessed on 15 March 2018) to explore the possible upstream miRNA regulators of Derlin-1 [32]. We identified the miR-375-3p (Figure 3A, TargetScan context++ score: −0.17) as one of the potential candidate miRNAs since Derlin-1 includes a predicted binding site at 3′-UTR of miR-375-3p. We found SV-HUC-1 cells had higher miR-375-3p expression compared with two UTUC cell lines (Figure 3B). We then examined the correlation between miR-375-3p and Derlin-1. In BFTC909 cells, the transfection of miR-375-3p did not change the transcription of *DERL1* estimated by real-time PCR (Figure 3C,D). Western blotting revealed that the overexpression of miR-375-3p notably reduced the protein expression of Derlin-1 when compared with the scrambled control (Figure 3E). As shown in Figure 3F, we performed dual-luciferase reporter assays to confirm whether miR-375-3p directly targeted Derlin-1. A remarkable decrease in luciferase activity was disclosed after the transfection with miR-375-3p mimics to wild-type Derlin-1 3′-UTR in BFTC909 cells. In contrast, there was no significant reduction of luciferase activity in the presence of mutated Derlin-1 3′-UTR as compared to the cells expressing scrambled control mimics (Figure 3G).

In addition, we investigated the expression level of both Derlin-1 mRNA and miR-375-3p in our tumor specimens (Figure 3H–J). Although not statistically significant, the muscle invasive stage (T2 or T3, n = 8) showed a trend of higher expression of Derlin-1 mRNA (*p* = 0.202) and lower expression of miR-375-3p (*p* = 0.839) compared with the non-muscle invasive stage (Ta or T1, n = 4). Taken together, these results implied that Derlin-1 may associate with a cancer aggressive phenotype. Moreover, miR-375-3p might directly target 3′-UTR of Derlin-1 and may inhibit the translation of oncogenic Derlin-1.

### 3.4. MiR-375-3p Inhibits Cell Migration and Invasion by Blocking EMT via Reducing Derlin-1 Expression

**Figure 4:**
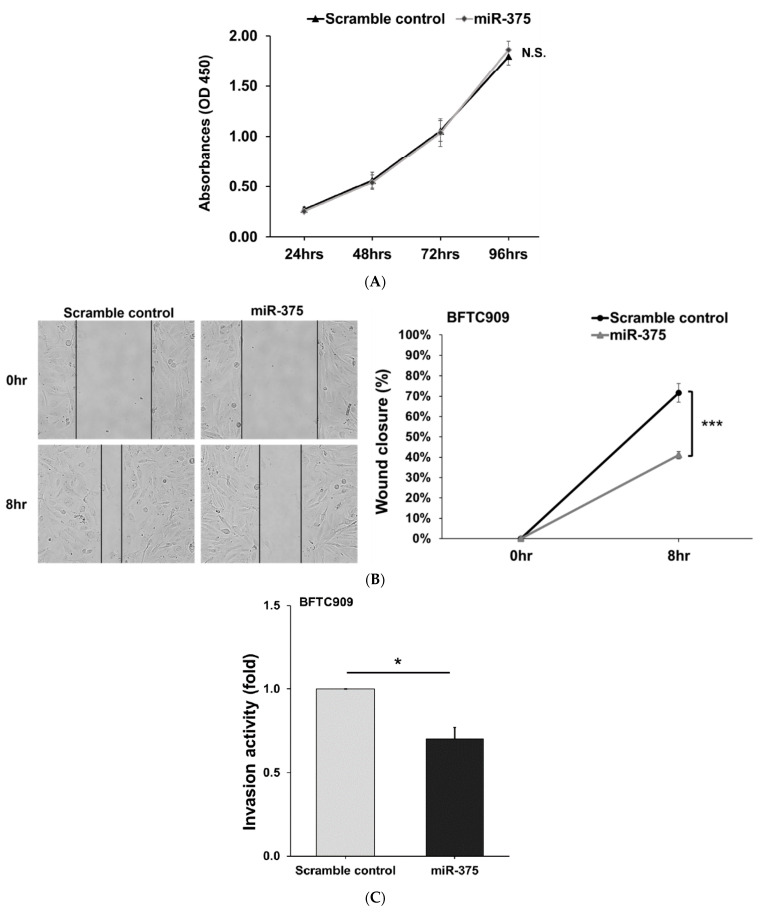
MiR-375 inhibits cell migration and invasion in the BFTC909 cell line. (**A**) MiR-375-3p upregulation did not affect the proliferation of BFTC909 cells (N = 3). (**B**) Upregulation of miR-375-3p represses migration of BFTC909 cells (N = 4). (**C**) MiR-375-3p overexpression represses invasion of BFTC909 cells (N = 3). Results were represented as mean ± SD; * *p* < 0.05 and *** *p* < 0.001. N.S: not significant.

The regulatory function of miR-375-3p was examined by cell proliferation assay, wound closure, and cell invasion assay in BFTC909 cells. The data showed that miR-375-3p overexpression notably inhibited both cell migration and invasion in BFTC909 cells, while no noticeable change in cell proliferation was found (Figure 4A–C).

**Figure 5:**
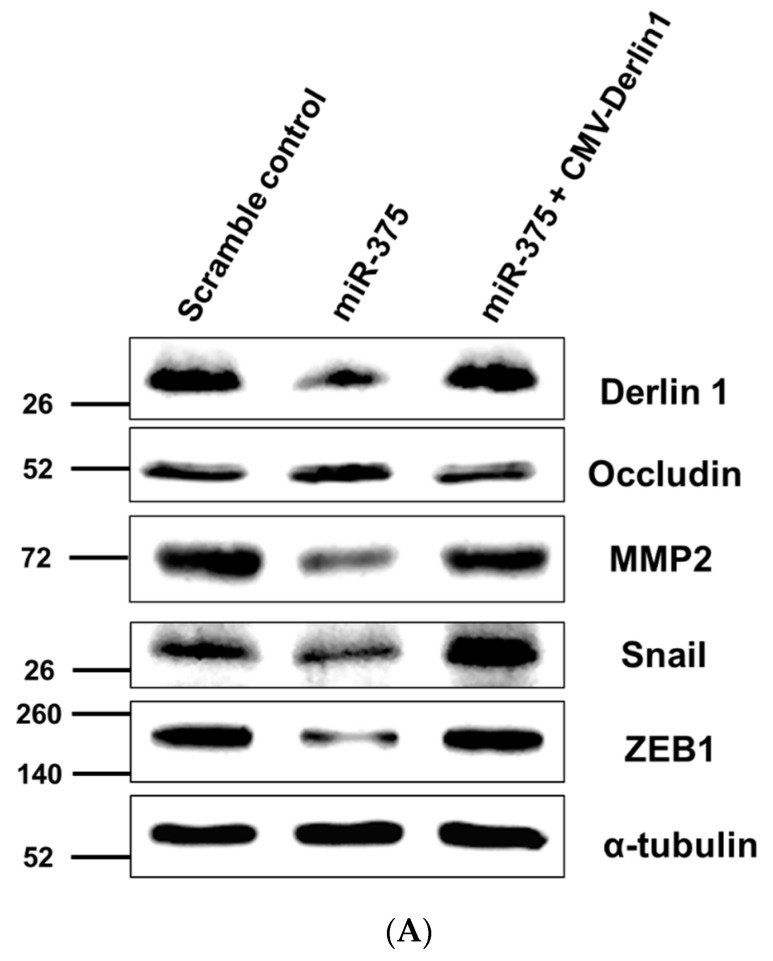

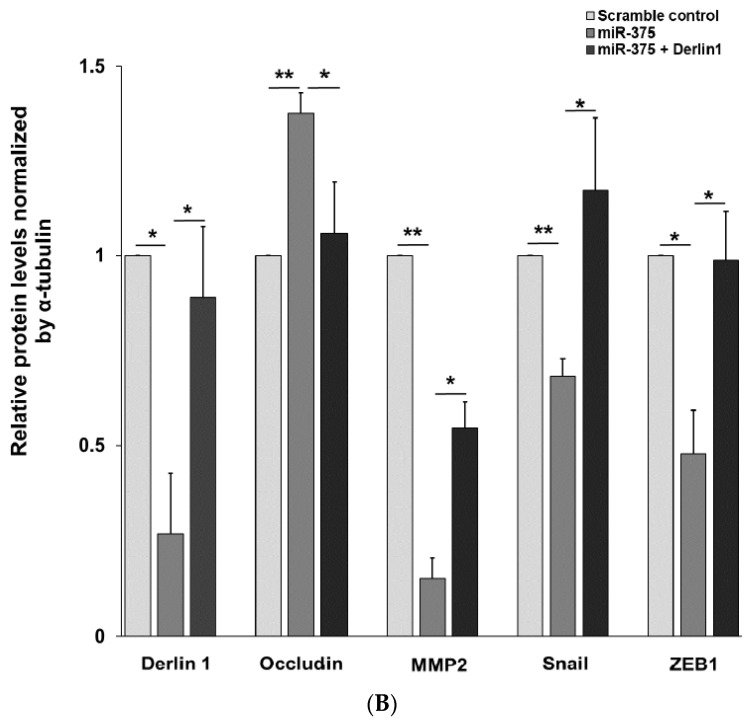

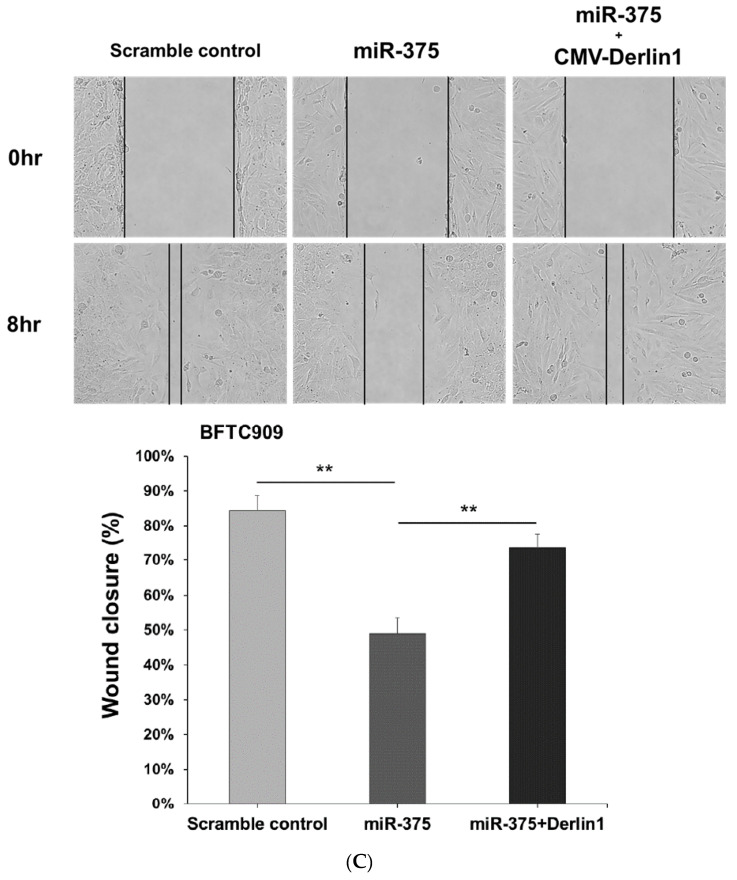

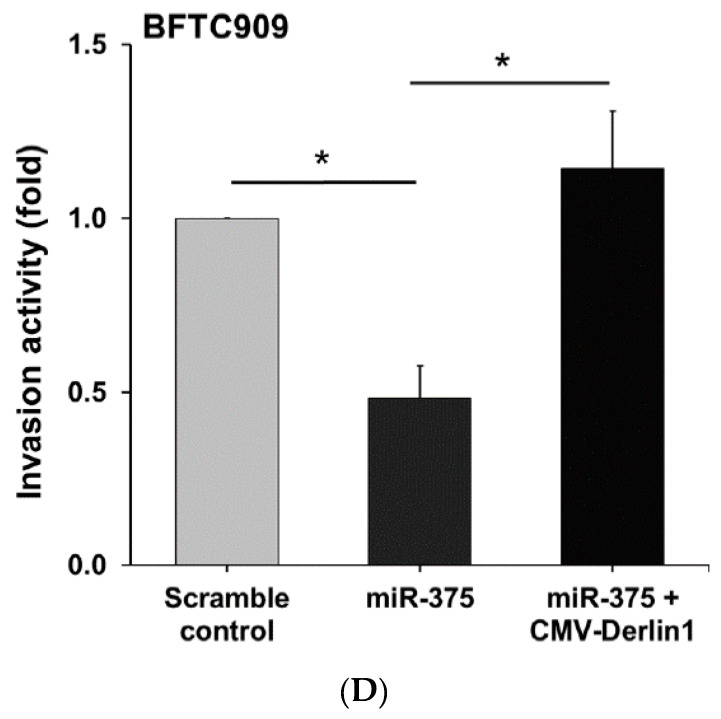
MiR-375-3p negatively regulates Derlin-1 and blocks EMT in BFTC909 cells. (**A**) Western blot revealed the restoration of Derlin-1, MMP-2, Snail, and ZEB1 after co-transfection of miR-375-3p mimics and CMV-Derlin-1 compared with cells transfected with miR-375-3p alone in BFTC909 cells with α-tubulin as a reference (**B**) Quantification of the protein levels of Derlin-1, occludin, MMP-2, Snail, and ZEB1 from (**A**) (N = 3). (**C**) miR-375-3p suppressed BFTC909 cell migration ability but restored by Derlin-1 overexpression (N = 3). (**D**) miR-375-3p repressed invasion of BFTC909 cells but restored by Derlin-1 overexpression (N = 3). Data were represented as mean ± SD; * *p* < 0.05, ** *p* < 0.01.

**Figure 6:**
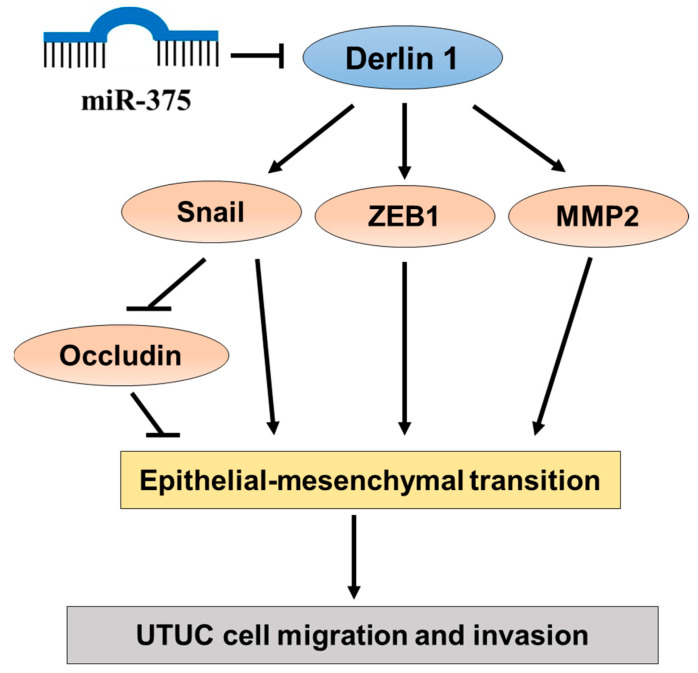
A schematic illustration of miR-375-3p/Derlin-1 mediated UTUC cell migration and invasion through EMT.

The regulatory effects of miR-375-3p on the well-known EMT markers, including occludin, Snail, MMP-2, and Zinc finger E-box binding homeobox 1 (ZEB1), were explored. The co-transfection of Derlin-1 overexpressing CMV vector was not repressed by miR-375-3p was performed to confirm its inhibitory function. Western blotting revealed the overexpression of miR-375-3p upregulated occludin and downregulated Snail, ZEB1, and MMP-2 protein levels. Moreover, the changes in the above EMT markers were reversed in response to the restoration of Derlin-1 (Figure 5A,B). Consistently, the upregulated expression of miR-375-3p co-transfected with Derlin-1 overexpression showed an enhanced migration and invasion ability in BFTC909 cells (Figure 5C,D). Taken together, these discoveries indicate that miR-375-3p suppresses cell invasion and migration by inversely regulating Derlin-1 and blocking EMT in UTUC cells (Figure 6).

## 4. Discussion

Little is known regarding the cellular and molecular mechanisms of UTUC because of its low prevalence worldwide [7]. In this study, for the first time, we implicated an oncogenic function of Derlin-1 in the progression of UTUC. Numerous studies have revealed that high expression of Derlin-1 is commonly observed in malignant tissues and less frequently in normal tissues in both humans and mice. Thus, Derlin-1 may have a clinical application as a novel cancer-targeting therapy [33]. Positive Derlin-1 expression has been detected in 66.7% and 70% of the breast carcinoma tissues and colon cancer tissues, respectively [23,26]. In patients with hepatocellular carcinoma, the expression rate of Derlin-1 was found to be 78.3% [22]. In human head and neck cancers, the positive expression rate of Derlin-1 was found to reach 94.7%, and 45.3% of positive tissues showing a high expression level of Derlin-1 [25]. In patients with bladder cancer, the positive Derlin-1 expression rate was 73.5% in muscle invasive urothelial carcinoma and 47.8% in non-invasive urothelial carcinoma [27]. Our results are in accordance with those of previous research, indicating a high expression level of Derlin-1 in UTUC tissues confers poor survival outcomes, indicating that Derlin-1 could act as an independent predictive factor in UTUC patients.

When solid tumor cells grow and divide, various stressful conditions, such as hypoxia and nutrient restriction, may occur and challenge tumor progression. The cancer-induced microenvironments, including hypoxia, inflammation, and angiogenesis, could trigger ER stress, which subsequently activates the unfolded protein response (UPR) and leads to cell apoptosis [34]. The cellular processes could be substantially impacted by these ER-intrinsic alterations and the ER stress in the tumor microenvironment (TME). The process is highly dynamic and can involve multiple pathways. In the past decade, numerous studies have focused on the roles of ER stress and UPR during tumorigenesis and attempted to uncover the possible pathways. Tumor growth and oncogenic transformation are multistep processes that may require some fundamental genetic alterations to drive ER stress in the TME. The overexpression of oncogenes or the loss of tumor suppressive genes might promote persistent protein synthesis and result in the overactivation of UPR. In view of this, the downstream signaling pathways of ER stress-related proteins have therefore been regarded as important modulators of cancer cell growth and metastasis and responders to chemotherapy, targeted therapy, and immunotherapy [34,35]. For instance, by driving ER stress and enhanced activation of the UPR, the oncogene MYC contributes to the genesis of a variety of human cancers [35]. Besides, mutant RAS is also a well-known oncogenic driver that takes responsibility for tumor initiation and progression via interplaying with UPR [35]. In normal epithelium cells, an intact UPR signal activated by the detection of misfolded proteins is critical for adaptation to any cause of ER stress. Upon tumorigenesis and cancer initiation, oncogenic stress in ER is now generally recognized as a UPR trigger [35,36]. So far, the mechanisms regarding how tumor cells adapt to long-term ER stress and overcome situations such as hypoxia, inflammation, and angiogenesis are still not fully understood. The oncogenic UPR signal pathways were reported to include PERK, IRE1α, and ATF6 pathways and perhaps helps cancer cells to overcome oncogenic stress [36]. For example, PERK activation might promote cancer cell survival through AKT signaling at a poor nutrition or hypoxia level. Furthermore, during tumor angiogenesis, VEGF also induces cancer cell progression via the activation of PERK and ATF6 pathways [36]. Therefore, the development of novel drugs to interfere with the ER stress-associated signaling pathways is imminent and promising.

Overexpression of some ER stress-related proteins, including Derlin-1, was examined in numerous tumor samples, and was highly correlated with tumor growth or metastasis [34,35]. In vitro, emerging researchers have suggested Derlin-1 might promote human cancer cell proliferation and migration, including adenocarcinoma of the colon [26], hepatocellular cancer [22], and head and neck cancer [25]. Compared to normal urothelium cells, a high level of Derlin-1 was detected in UTUC cells in our study. We further demonstrated that downregulation of Derlin-1 represses tumor cell invasion and migration rather than cell proliferation in UTUC. However, the specific molecular mechanism of Derlin-1 in cancer progression remains unclear and might vary among different types of malignancy. In human breast cancer, Derlin-1 was reported to protect cancer cells by eliminating ER stress-induced apoptosis and further contribute to the metastatic properties in cancer cells [23]. Different with our study, Derlin-1 may involve regulation of the PI3K/AKT pathway and contribute to cell proliferation in both colon cancer [26] and hepatocellular carcinoma [22]. In non-small cell lung carcinoma, Derlin-1 may also promote cancer cell invasion through the ERK/MMP pathway [37]. Taken together, this evidence suggests the protection role of Derlin-1 expression with regard to stresses encountered during cancer cell growth and the process of tumorigenesis.

The mechanism by which Derlin-1 regulates cancer progression still needs further investigation, including more in vitro experiments and in vivo studies. Previous research has reported that miR-598, miR-132, and miR-181d could target Derlin-1 and regulate tumor invasion, migration, or metastasis [28,38,39]. To validate the underlying mechanism of Derlin-1 in UTUC, we further verified *DERL1* as a direct target gene of miR-375-3p. More importantly, the present research demonstrated that miR-375-3p negatively regulated Derlin-1 to inhibit the migration and invasion of UTUC cells through EMT, confirming the inhibitory role of miR-375-3p in UTUC. Earlier studies have suggested miR-375-3p plays an anti-oncogenic role by suppressing cell growth and invasion in fibrolamellar carcinoma [40] or enhancing the chemosensitivity of 5-fluorouracil via targeting thymidylate synthase in colon cancer [41]. Downregulation of miR-375-3p was verified to be associated with aggressive behavior in oral cancer [14]. Besides, in hepatoma cells, miR-375-3p may repress tumor angiogenesis and reverse resistance to target therapy [42]. In colorectal cancer cells, by targeting YAP1 and SP1, miR-375-3p was found to repress tumorigenesis and partially modulate the chemoresistance of 5-fluorouracil [43]. On the contrary, miR-375-3p has also been identified as an oncogene in colorectal cancer because of its ability to promote invasion and migration by targeting RECK [44]. In an integrated analysis of colorectal cancer microRNA datasets, downregulated miR-375-3p was one of the most frequently found miRNAs within the analyzed datasets [15]. These conflicting findings suggest that the correlation of miR-375-3p and cancer cells is still controversial. Although miR-375-3p is regarded as a tumor-suppressive miRNA in numerous tumors, the molecular mechanism of miR-375-3p in UTUC remains unclear.

In this research, our data showed that miR-375-3p notably inhibited cell invasion and migration in UTUC cells. Western blotting analysis has revealed that Derlin-1 knockdown decreases Snail expression in UTUC cells. MiR-375-3p overexpression also decreases the EMT markers, including Snail, MMP-2, and ZEB1. Moreover, these effects of repressing Snail, MMP-2, and ZEB1 were restored by Derlin-1 overexpression, implying that Derlin-1 may activate the EMT process by regulating these known transcriptional factors and matrix metalloproteinases in UTUC cells. EMT is an important process that induces aggressiveness in malignant cells, and the development, progression, and metastasis of urothelial carcinoma are associated with the involvement of EMT [45,46]. Previous research has also established the significance and implications of urothelial cancer cell migration and invasion via known regulators of EMT such as Snail, MMP-2, and ZEB1 [31]. Snail is a famous inducer and a prominent molecular marker in the development and progression of UTUC [47]. In the initial invasion stage, tumor cells need the MMP family to destruct the surrounding matrix components and facilitate tumor spreading. Hence, many studies have focused on the effects of MMP-2 on cancer invasion and migration. Increased expression of MMP-2 was described to be correlated with intravasation and lymph node metastasis of tumor cells into the blood vessels [48]. ZEB1 is another well-known inducer that promotes tumor invasion and migration by inducing EMT in carcinoma cells and is involved in metastasis and therapy resistance in cancers, including urothelial carcinoma [49]. The function of the occludin protein is to maintain epithelial cell polarity, and Snail may directly bind to the E-boxes of the promoters of the occludin gene, resulting in repression of occludin protein level [50]. In this study, silencing Derlin-1 did not regulate occludin expression in BFTC909 cells (Figure 2F). Overexpression of Derlin-1 reduces the protein level of occludin in UM-UC-14 cells (Figure 2G). We propose that Derlin-1 may act as an intermediate regulator, affecting the expression of occludin according to cell specificity or partially regulating by Snail. It needs to find more UTUC cells to verify in the future. The miRNA-mediated regulation of EMT is a highly integrated process [51]. Our study indicates that miR-375-3p might reduce the protein levels of MMP-2, ZEB1 and Snail via targeting Derlin-1 and eventually repress the cell migration and invasion of UTUC. More research exploring these issues will provide useful information for the widespread comprehension of the molecular mechanisms of tumors.

There were several limitations in our study. First, we did not perform experiments in an animal model to support our results in vitro. Second, the major functional experiments were performed on a single UTUC cell line, BFTC909. One of the reasons is that our previous study indicated that BFTC909 had higher invasive ability than UM-UC-14 cells [29]. Besides, BFTC909 was established from the primary UTUC of patients from a blackfoot disease endemic area in Taiwan, which is more compatible with our patient cohort. Third, we did not examine some famous EMT markers, such as the Twist and claudin family. We believe our experimental data in EMT is relatively sufficient, but more experiments will provide more comprehensive evidence. Fourth, the expression levels of Derlin-1 and miR-375-3p were only examined in only 12 tissue samples to compare the difference between different tumor stages. There was no significant association between the expression of Derlin-1 mRNA and miR-375-3p in our tumor specimens (Figure 3J). This result is actually compatible with our findings in cell line experiments that overexpression of miR-375-3p did not change the transcription of *DERL1* but reduced the Derlin-1 protein expression (Figure 3D,E). However, we did not examine the protein expression levels in our specimens to confirm this hypothesis. Fifth, we did not compare the expressions of Derlin-1 and miR-375-3p in cancer tissue and nearby normal tissue from patients with UTUC. More experiments with tissue samples and the analysis of circulating Derlin-1 or miR-375-3p in a larger series of patients is mandatory to confirm the results obtained in our study and provide promising strategies for future translation to the clinical setting.

## 5. Conclusions

Our study demonstrated that miR-375-3p suppresses invasion and migration by directly targeting Derlin-1 in UTUC cells, suggesting Derlin-1 may act as a useful predictor of prognosis and potential therapeutic target in patients with UTUC. Moreover, miR-375-3p could be incorporated into the UTUC molecular pathway. MiR-375-3p overexpression could inversely regulate the protein levels of EMT markers, suggesting that miR-375-3p functions as a tumor suppressive microRNA to block EMT by directly binding to Derlin-1 in UTUC cells.

## Figures and Tables

**Table 1:** Association of Derlin-1 with clinicopathological characteristics in UTUC.

			Derlin-1				
			Low		High		
Variables	Item	Patient No.	No.	%	No.	%	*p* Value
Overall		100	48	48	52	52	
Stage	I/II	61	35	72.9	26	50	0.019 ^#^
	III/IV	39	13	27.1	26	50	
Grade	Low	22	13	27.1	9	17.3	0.238 ^#^
	High	78	35	72.9	43	82.7	
Gender	Female	58	28	58.3	30	57.7	0.948 ^#^
	Male	42	20	41.7	22	42.3	
Age (years)	<65	34	17	35.4	17	32.7	0.774 ^#^
	≥65	66	31	64.6	35	67.3	
Tumor size (cm)	<2	20	12	25	8	15.4	0.230 ^#^
	≥2	80	36	75	44	84.6	
LN Metastasis	No	87	44	91.7	43	82.7	0.182 ^##^
	Yes	13	4	30.8	9	17.3	
Location	Renal pelvis	50	22	45.8	28	53.8	0.643 ^#^
	Ureter	39	21	43.8	18	34.6	
	Both	11	5	10.4	6	11.5	
Tumor number	Single	89	43	89.6	46	88.5	1.000 ^#^
	Multiple	11	5	10.4	6	11.5	
Distant Metastasis	Negative	82	44	91.7	38	73.1	0.019 ^##^
	Positive	18	4	8.3	14	26.9	
Bladder recurrence	Negative	74	41	85.4	33	63.5	0.012 ^#^
	Positive	26	7	14.6	19	36.5	
Local recurrence	Negative	78	44	91.7	34	65.4	0.002 ^##^
	Positive	22	4	8.3	18	34.6	
Cancer Death	No	83	46	95.8	37	71.2	0.001 ^##^
	Yes	17	2	4.2	15	28.8	

^#^ The *p* value was counted by chi-square test. ^##^ The *p* value was counted by Fisher’s test.

**Table 2:** Analysis of bladder recurrence-free survival for UTUC.

Variables	Item	Univariate			Multivariable		
		HR	95% CI	*p* Value	HR	95% CI	*p* Value
Stage	III/IV	1.2	(0.54, 2.66)	0.661	-	-	-
	I/II	1			-		
Grade	High	0.91	(0.37, 2.28)	0.845	-	-	-
	Low	1			-		
Gender	Male	1.21	(0.56, 2.61)	0.63	-	-	-
	Female	1			-		
Age (years)	≥65	1.75	(0.76, 4.07)	0.191	1.98	(0.84, 4.67)	0.117
	<65	1			1		
Tumor number	Multiple	1.97	(0.58, 6.65)	0.275	2.97	(0.83, 10.60)	0.093
	Single	1			1		
Derlin-1	High	3.27	(1.37, 7.80)	0.008	3.62	(1.50, 8.76)	0.004
	Low	1			1		

**Table 3:** Analysis of progression-free survival for UTUC.

Variables	Item	Univariate			Multivariable		
		HR	95% CI	*p* Value	HR	95% CI	*p* Value
Stage	III/IV	6.72	(2.41, 18.68)	<0.001	4.81	(1.65, 14.06)	0.004
	I/II	1			1		
Grade	High	6.86	(0.92, 51.13)	0.06	4.42	(0.56, 34.60)	0.157
	Low	1			1		
Gender	Male	0.8	(0.34, 1.91)	0.618	-	-	-
	Female	1			-		
Age (years)	≥65	1.33	(0.54, 3.26)	0.539	-	-	-
	<65	1			-		
Tumor number	Multiple	2.16	(0.63, 7.47)	0.222	2.78	(0.76, 10.14)	0.121
	Single	1			1		
Derlin-1	High	5.07	(1.71, 15.00)	0.003	4.68	(1.52, 14.44)	0.007
	Low	1			1		

**Table 4:** Analysis of cancer-specific survival for UTUC.

Variables	Item	Univariate			Multivariable		
		HR	95% CI	*p* Value	HR	95% CI	*p* Value
Stage	III/IV	7.07	(2.22, 22.51)	0.001	5.65	(1.75, 18.26)	0.004
	I/II	1			1		
Grade	High	2.38	(0.54, 10.40)	0.25	1.52	(0.30, 7.75)	0.615
	Low	1			1		
Gender	Male	1.11	(0.43, 2.88)	0.837	-	-	-
	Female	1			-		
Age (years)	≥65	1.94	(0.63, 5.97)	0.246	2.92	(0.87, 9.77)	0.082
	<65	1			1		
Tumor number	Multiple	2.54	(0.73, 8.87)	0.145	2.98	(0.80, 11.12)	0.104
	Single	1			1		
Derlin-1	High	7.81	(1.78, 34.16)	0.006	6.24	(1.39, 27.97)	0.017
	Low	1			1		

## Data Availability

Data are contained within the article.

## Supplementary Materials

The following are available online at https://www.mdpi.com/article/10.3390/cancers14040880/s1, File S1: Original Western blots.

## Abbreviations

3′-untranslated regions, 3′-UTR; Ak strain transforming, protein kinase B, AKT; Bioresource Collection and Research Center, BCRC; confidence interval, CI; cytomegalovirus, CMV; Dulbecco’s Modified Eagle Medium, DMEM; European Collection of Authenticated Cell Cultures, ECACC; Eagle’s Minimum Essential Medium, EMEM; epithelial–mesenchymal transition, EMT; endoplasmic reticulum, ER; extracellular signal-regulated kinase, ERK; fetal bovine serum, FBS; hazard ratio, HR; immunoglobulin G, IgG; microRNAs, miRNAs; matrix metallopeptidase 2, MMP-2; messenger RNA, mRNA; National Comprehensive Cancer Network, NCCN; nonessential amino acids, NEAA; phosphatidylinositol 3-kinases, PI3K; radical nephroureterectomy, RNU; reverse transcription polymerase chain reaction, RT-PCR; tumor microenvironment, TME; unfolded protein response, UPR; upper tract urothelial carcinoma, UTUC; World Health Organization, WHO; Zinc Finger E-Box Binding Homeobox 1, ZEB1.
