# Supplementary material for: MicroRNA-375-3p Suppresses Upper Tract Urothelial Carcinoma Cell Migration and Invasion via Targeting Derlin-1
Source: Cancers (Basel). 2022 Feb 10;14(4):880. doi: 10.3390/cancers14040880 (PMC8869792; doi:10.3390/cancers14040880)
Supplement: Supplementary file 1 [file cancers-14-00880-s001.zip › cancers-1563148-supplementary.pdf]

MicroRNA-375-3p suppresses upper tract urothelial carcinoma cell migration and invasion via targeting Derlin-1

Jhen-Hao Jhan [1, 2, 3, 4], Wei-Chi Hsu [1, 5], Yi-Chen Lee [6], Wei-Ming Li [1, 2, 7], A-Mei Huang [4, 5, 8], Hui-Hui Lin [1, 2], Chien-Sheng Wang [2, 4], Yi-Ru Wu [9], Ching-Chia Li [1, 2], Wen-Jeng Wu [1, 2, 5], Hung-Lung Ke [1, 2, 5, 10]

[1] Department of Urology, School of Medicine, College of Medicine, Kaohsiung Medical University, Kaohsiung, Taiwan.
[2] Department of Urology, Kaohsiung Medical University Hospital, Kaohsiung Medical University, Kaohsiung, Taiwan.
[3] Department of Urology, Kaohsiung Municipal Siaogang Hospital, Kaohsiung, Taiwan
[4] Graduate Institute of Clinical Medicine, College of Medicine, Kaohsiung Medical University, Kaohsiung, Taiwan.
[5] Graduate Institute of Medicine, College of Medicine, Kaohsiung Medical University, Kaohsiung, Taiwan.
[6] Department of Anatomy, School of Medicine, College of Medicine, Kaohsiung Medical University, Kaohsiung, Taiwan.
[7] Department of Urology, Ministry of Health and Welfare Pingtung Hospital, Pingtung, Taiwan.
[8] Department of Biochemistry, School of Medicine, College of Medicine, Kaohsiung Medical University, Kaohsiung, Taiwan.
[9] General Division, Kaohsiung Medical University Hospital, Kaohsiung Medical University, Kaohsiung, Taiwan.
[10] Department of Urology, Kaohsiung Municipal Ta-Tung Hospital, Kaohsiung Medical University Hospital, Kaohsiung Medical University, Kaohsiung, Taiwan.

Corresponding author: Hung-Lung Ke, M.D., Ph.D., Department of Urology, School of Medicine, College of Medicine, Kaohsiung Medical University, Kaohsiung, Taiwan, 100, Shih-Chuan 1st Road, Kaohsiung, 80708, Taiwan., E-mail: hunglungke@gmail.com, telephone number: +886 07-3121101 #6694. Fax number: +886 07-3211033.

File S2.A

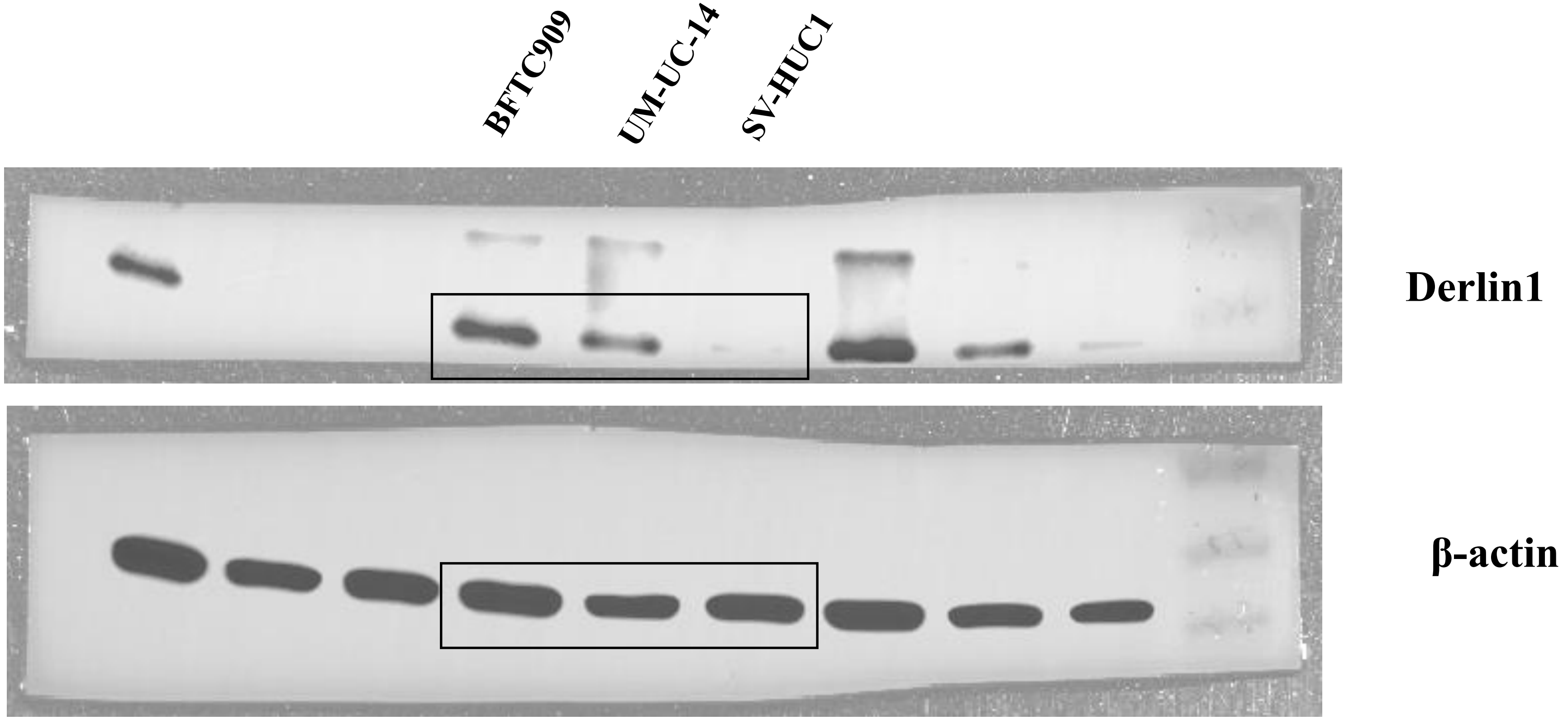

File S2.A (S2.A): Full length images of blots for Figure 2A in main paper. Dotted rectangles indicate the regions used in the figures. PVDF membranes were used for probing with the respective primary antibody.

File S2.B

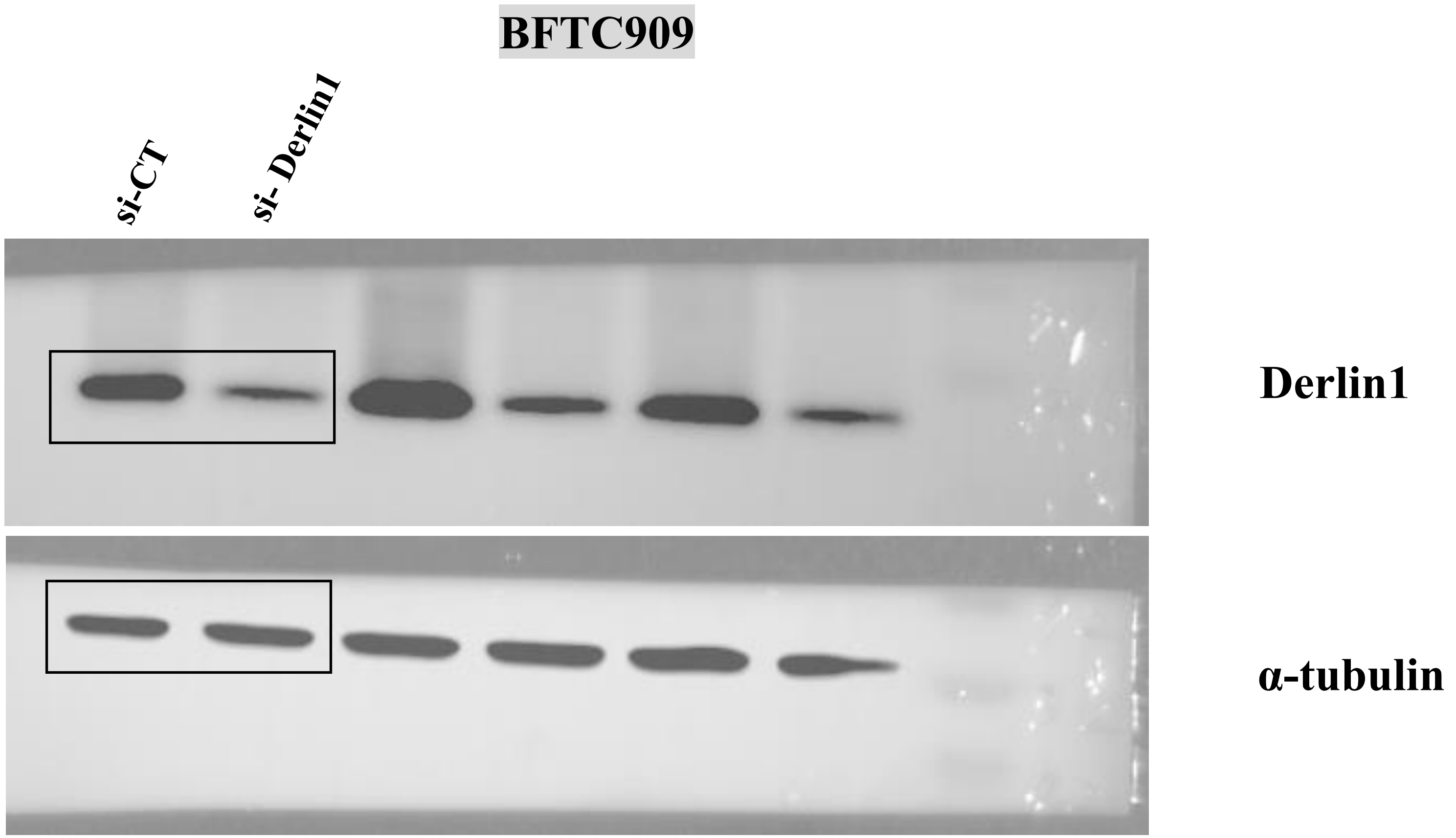

File S2.B (S2.B): Full length images of blots for Figure 2B in main paper. Dotted rectangles indicate the regions used in the figures. PVDF membranes were used for probing with the respective primary antibody.

File S2.F **BFTC909**

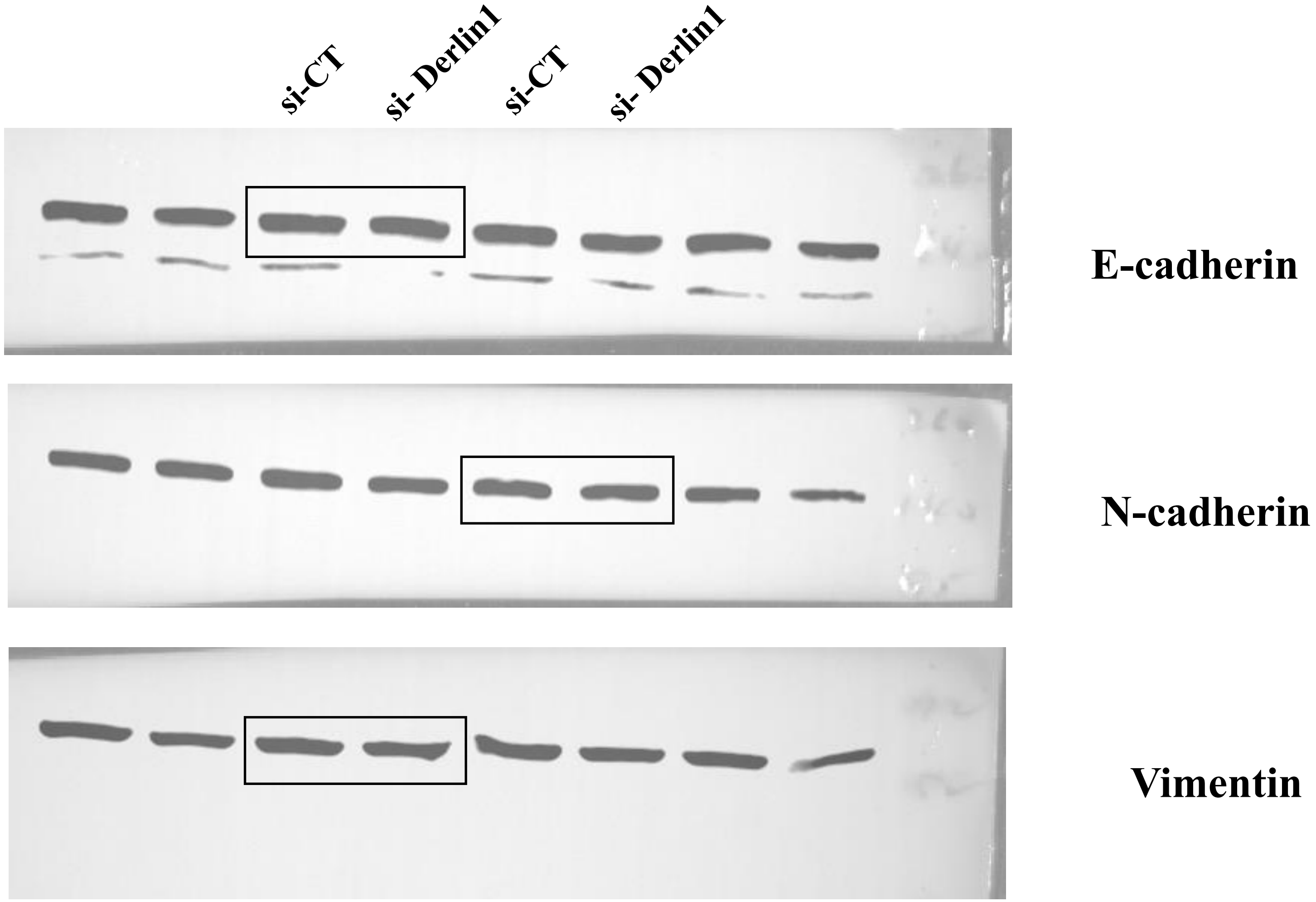

File S2.F (S2.F): Full length images of blots for Figure 2F in main paper. Dotted rectangles indicate the regions used in the figures. PVDF membranes were used for probing with the respective primary antibody.

File S2.F BFTC909

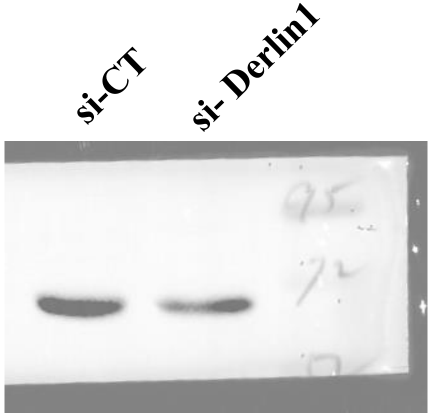

MMP2

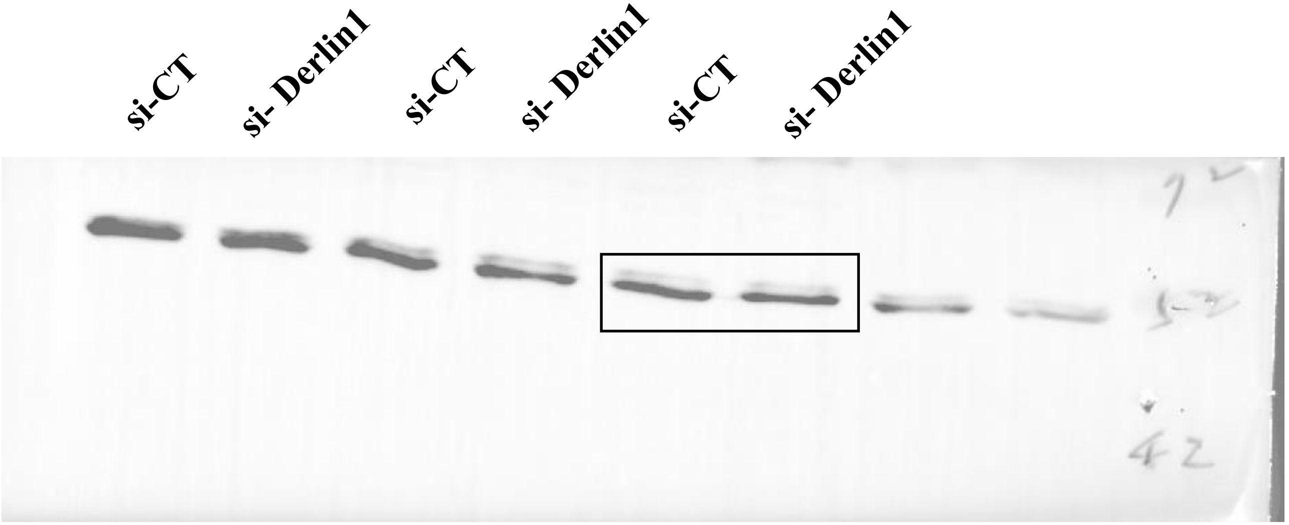

Occludin

File S2.F (S2.F): Full length images of blots for Figure 2F in main paper. Dotted rectangles indicate the regions used in the figures. PVDF membranes were used for probing with the respective primary antibody.

File S2.F BFTC909

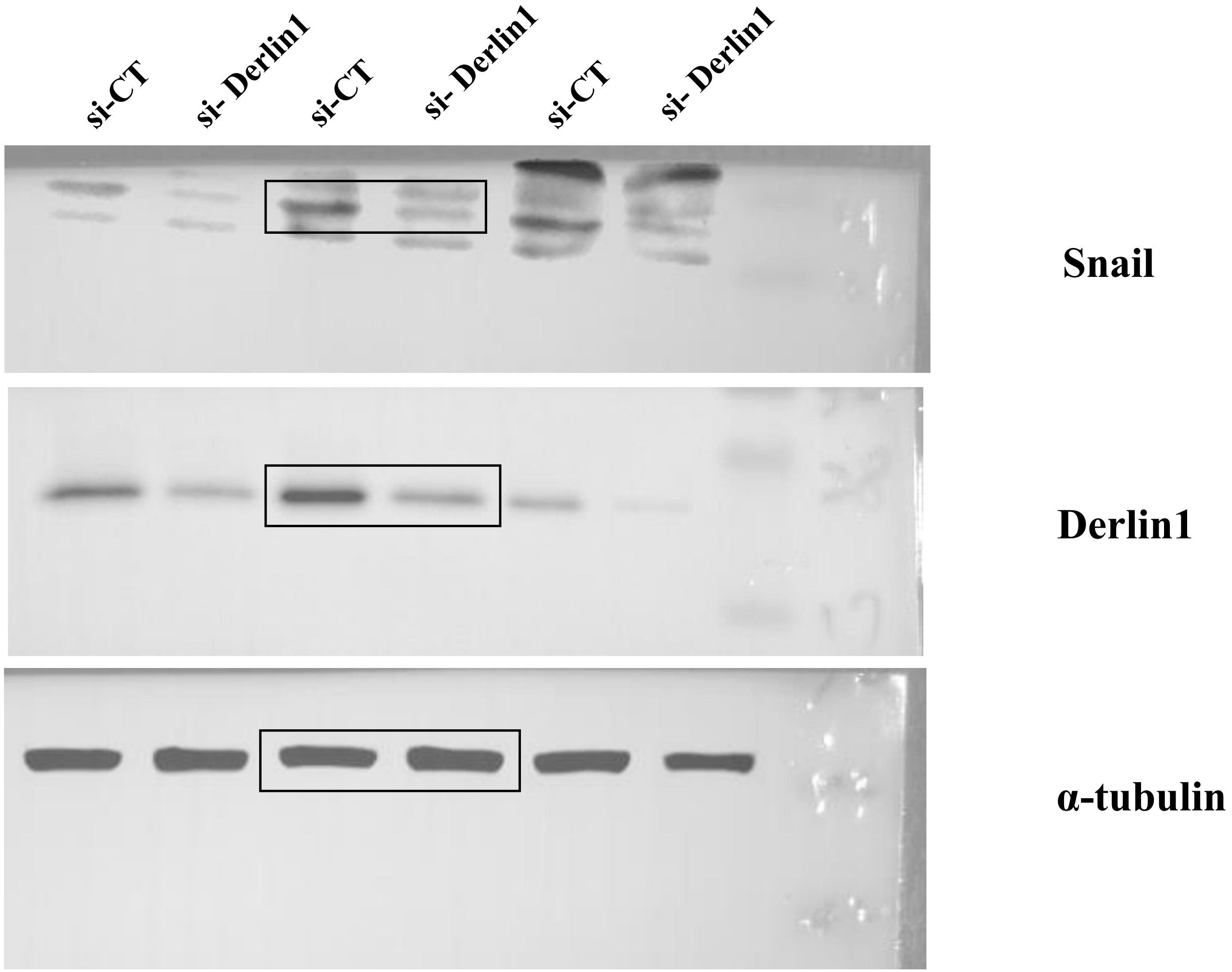

File S2.F (S2.F): Full length images of blots for Figure 2F in main paper. Dotted rectangles indicate the regions used in the figures. PVDF membranes were used for probing with the respective primary antibody.

File S2.G UM-UC-14

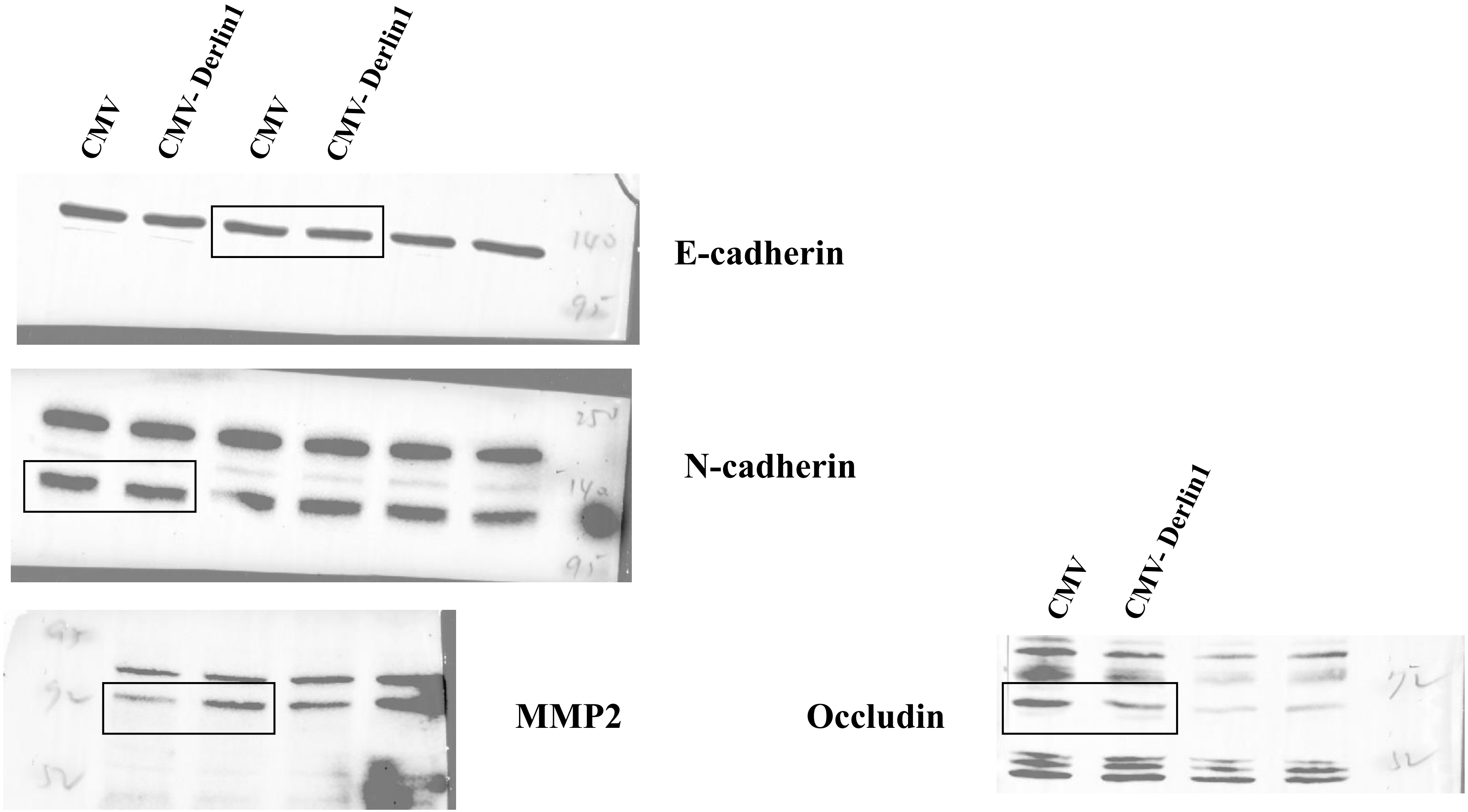

File S2.G (S2.G): Full length images of blots for Figure 2G in main paper. Dotted rectangles indicate the regions used in the figures. PVDF membranes were used for probing with the respective primary antibody.

File S2.G UM-UC-14

CMV CMV- Derlin1

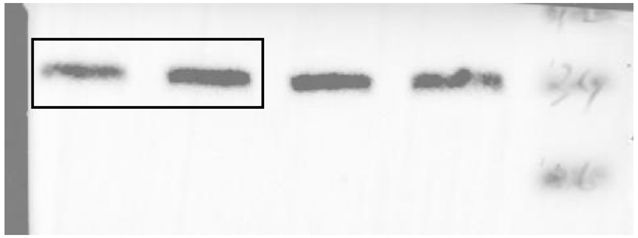

Snail

CMV CMV- Derlin1

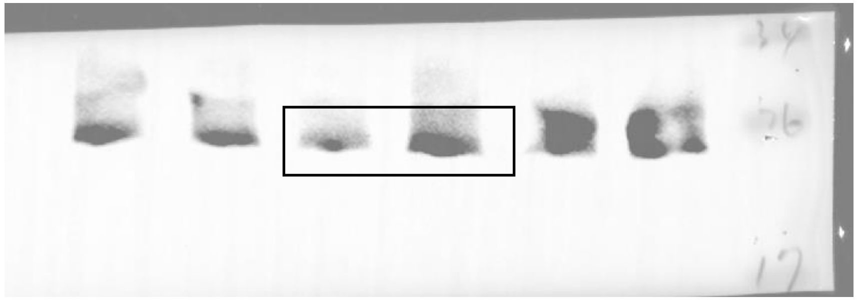

Derlin1

CMV CMV- Derlin1

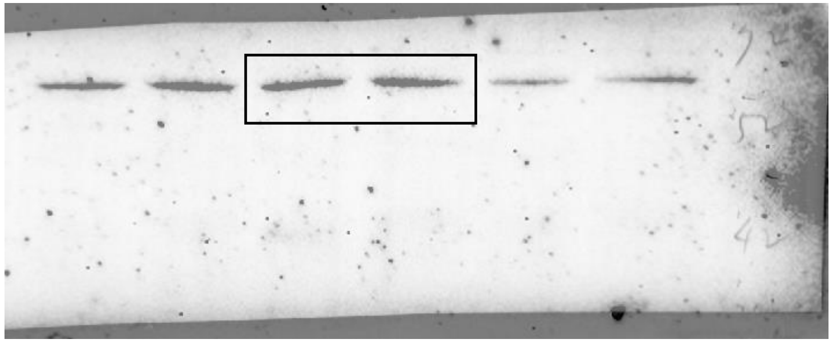

Vimentin

File S2.G (S2.G): Full length images of blots for Figure 2G in main paper. Dotted rectangles indicate the regions used in the figures. PVDF membranes were used for probing with the respective primary antibody.

File S2.G **UM-UC-14**

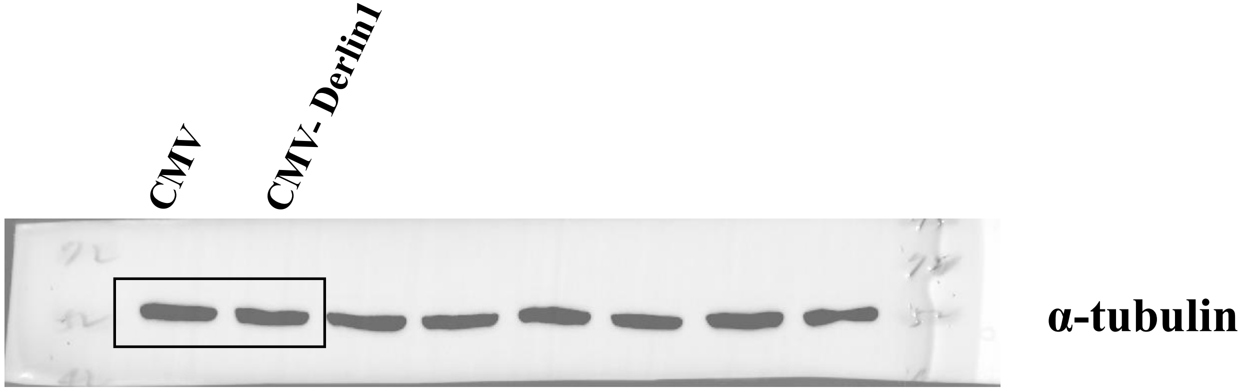

File S2.G (S2.G): Full length images of blots for Figure 2G in main paper. Dotted rectangles indicate the regions used in the figures. PVDF membranes were used for probing with the respective primary antibody.

File S3.D **BFTC909**

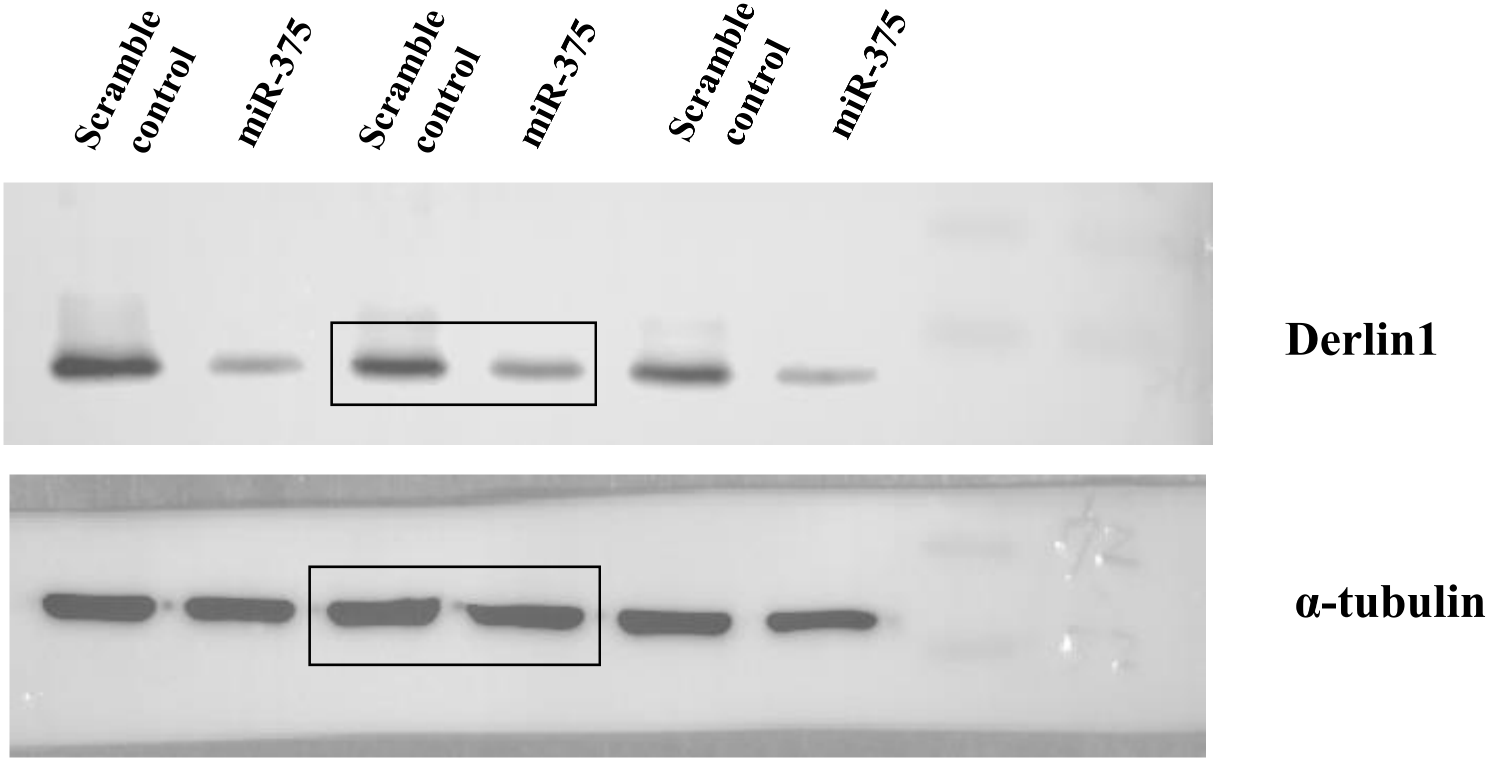

File S3.D (S3.D): Full length images of blots for Figure 3D in main paper. Dotted rectangles indicate the regions used in the figures. PVDF membranes were used for probing with the respective primary antibody.

File S5.A BFTC909

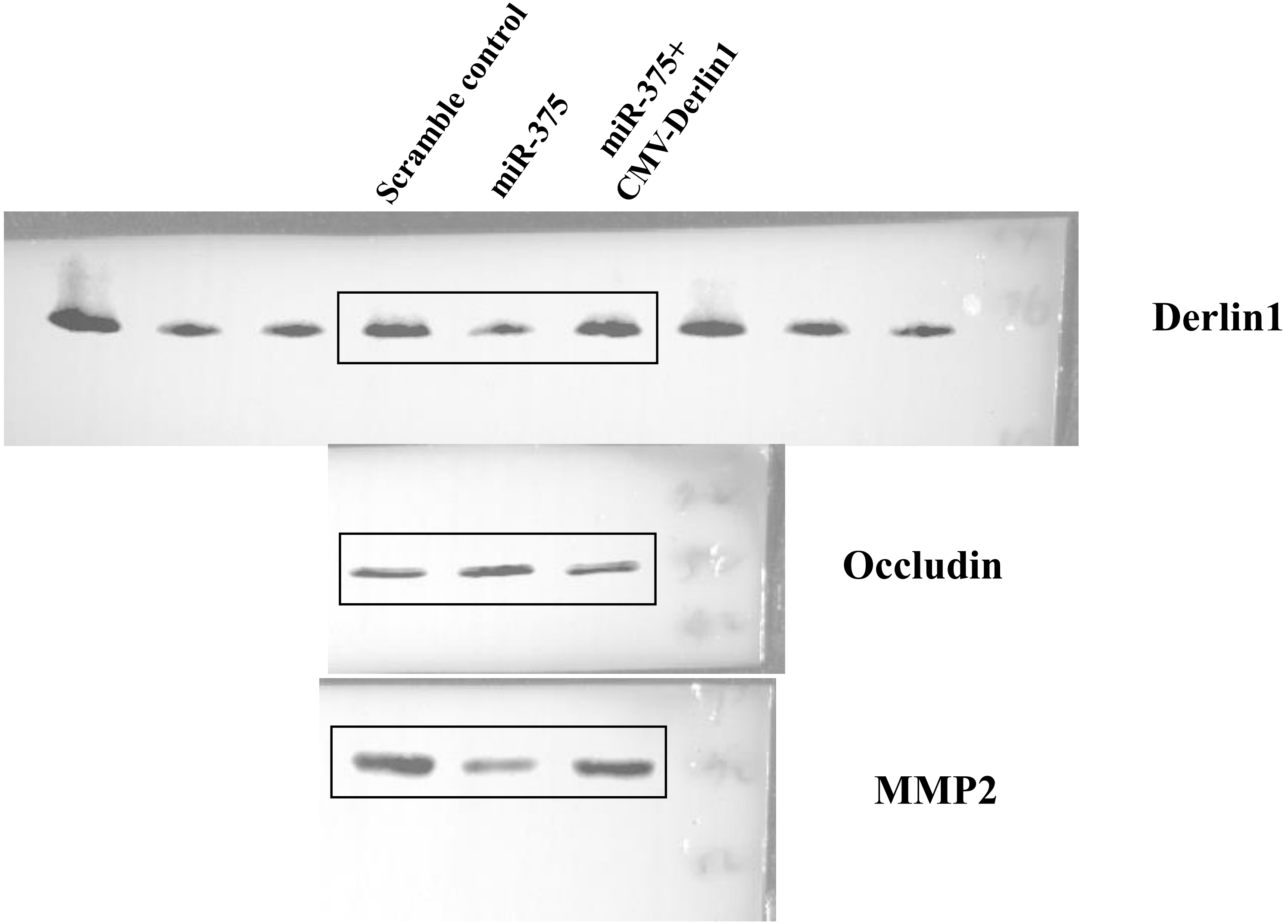

File S5.A (S5.A): Full length images of blots for Figure 5A in main paper. Dotted rectangles indicate the regions used in the figures. PVDF membranes were used for probing with the respective primary antibody.

File S5.A **BFTC909**

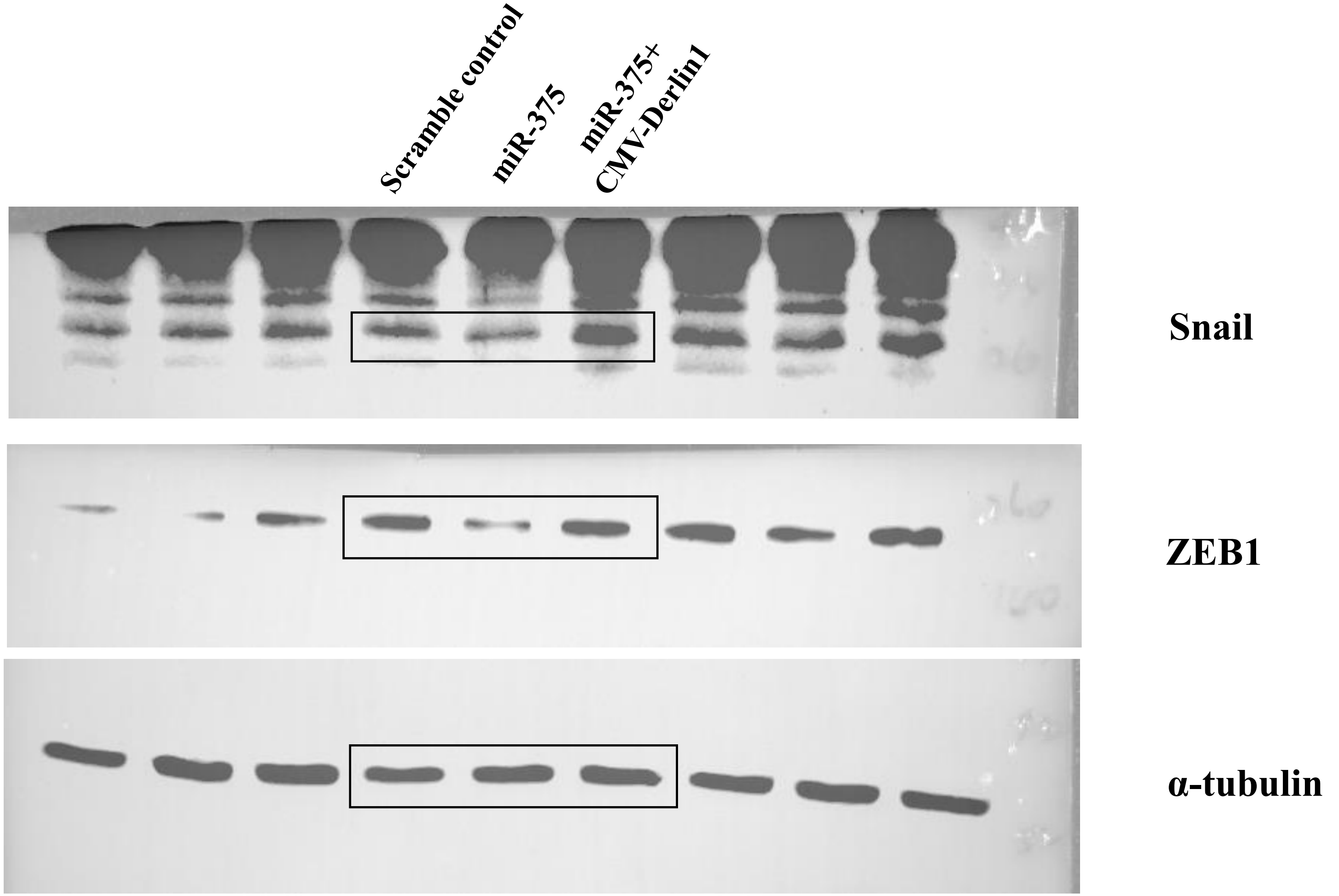

File S5.A (S5.A): Full length images of blots for Figure 5A in main paper. Dotted rectangles indicate the regions used in the figures. PVDF membranes were used for probing with the respective primary antibody.
